# Synthesis of Nonadentate Ligand Diethylene Glycol-Bis(3-Aminopropyl Ether)-*N*,*N*,*N′*,*N′*-Tetraacetic Acid DEGTA and Its Complexation Behavior toward Trivalent Lanthanides and Actinides

**DOI:** 10.1021/acs.inorgchem.4c05049

**Published:** 2025-02-28

**Authors:** Sebastian Friedrich, Adrian Näder, Björn Drobot, Jerome Kretzschmar, Thorsten Stumpf, Astrid Barkleit

**Affiliations:** Institute of Resource Ecology, Helmholtz-Zentrum Dresden−Rossendorf, Dresden 01328, Germany

## Abstract

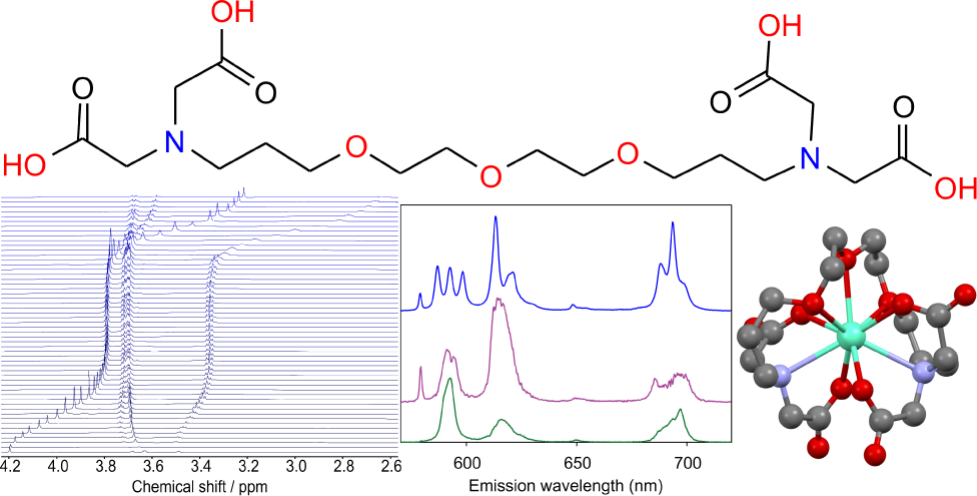

A new nonadentate ligand, DEGTA (diethylene glycol-bis(3-aminopropyl ether)-*N*,*N*,*N′*,*N′*-tetraacetic acid), from the polyaminopolycarboxylate family, was synthesized in a two-step reaction. The ligand’s pH-dependent behavior (structure and p*K*_a_ values) was determined by nuclear magnetic resonance (NMR) spectroscopy. The complexation ability of the ligand toward trivalent lanthanides and actinides was studied by time-resolved laser-induced fluorescence spectroscopy (TRLFS) using Eu(III) and Cm(III) as representatives. For Eu(III), two species occurring at different pH values were observed and corroborated by concentration- and pD-dependent NMR-titration series, viz. [EuH_2_(DEGTA)]^+^ and [Eu(DEGTA)]^−^. The latter is shown to be nine-coordinate, forming isostructural complexes with Cm(III) and Sm(III) as inferred from TRLFS and 2D NMR experiments, respectively. Since DEGTA can be seen as a consecutive derivative of EDTA and EGTA with an elongated backbone, the structures of their Eu(III) complexes were calculated using density functional theory (DFT) and the same aminoacetate binding motif proven by Fourier-transform infrared (FT-IR) spectroscopy. Upon comparison of structure–property relationships (denticity and chain length vs coordination geometry and complex stability) one can draw conclusions on DEGTA’s complexation behavior in particular, and some generalizable trends in complexation properties within the complexone series are discussed. Looking further ahead, this knowledge will help in further developing decontamination, decommissioning, and decorporation strategies.

## Abstract 2

We synthesized a new nonadentate aminopolycarboxylate ligand (L) and examined its complexation behavior toward selected trivalent lanthanides and actinides, M(III), by comprehensive multispectroscopic pH- and concentration-dependent experiments complemented by DFT calculations. Structure and speciation analyses reveal isostructural complexes of the type [M(III)H_2_L]^+^ and [M(III)L]^−^, predominating at low and high pH, respectively, where the trivalent f elements display complete (nine-fold) coordination exclusively by L without water ligands.

## 1 Introduction

**Figure 1:**
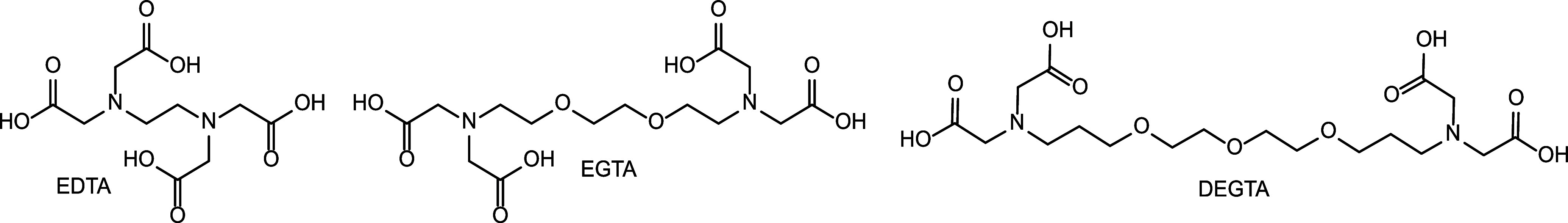
Structures of the hexadentate ligand EDTA (left), octadentate ligand EGTA (middle), and nonadentate ligand DEGTA (right).

Complexones from the aminopolycarboxylate family, like tetradentate nitrilotriacetate (NTA), hexadentate ethylenediaminetetraacetate (EDTA), and octadentate ethylene glycol-bis(2-aminoethyl ether)-*N*,*N*,*N’*,*N’*-tetraacetate (EGTA), have been shown to possess a high affinity toward trivalent lanthanides, Ln(III), as well as actinides, An(III).^1^ Some representatives of this family, especially NTA and EDTA, are already commonly used in the nuclear industry in terms of decontamination along with the decommissioning of nuclear power plants.^2,3^ Furthermore, these two representatives besides another related ligand (diethylenetriaminepentaacetic acid, DTPA) have been tested or are in use as decorporation agents as treatment against plutonium, americium, and curium incorporation.^4−16^ For further development of decommissioning as well as decorporation strategies, the metal–ligand interactions have to be well understood. Therefore, upon extending this series and understanding trends in complexation behavior among the series, the nonadentate ligand DEGTA was synthesized. The aim was to examine the type and number of coordinating atoms as well as the stability of the formed complexes. Consequently, the backbone was modified rendering the ligand to possess three more potential binding sites than EDTA and one more than EGTA, respectively (see Figure 1).

Based on a previously reported workflow to achieve robust thermodynamic data, the complementary methods nuclear magnetic resonance (NMR) spectroscopy and time-resolved laser-induced fluorescence spectroscopy (TRLFS) were combined.^1^ To provide accurate thermodynamic data, precise acid dissociation constants are necessary. They have been determined by means of ^1^H NMR spectroscopic pH-titration and further used to determine stability constants of the complexes forming with trivalent europium, Eu(III), and curium, Cm(III), employing TRLFS using excellent luminescence properties allowing experiments at metal concentrations as low as 10 and 0.3 μM, respectively. Information regarding complex structure was obtained from FT-IR and, particularly, NMR spectroscopy complemented by DFT calculation. With NMR, the Eu(III) system was comprehensively examined in pD- and concentration-dependent series and supplemented by DEGTA complexation of diamagnetic La(III) and slightly paramagnetic Sm(III).

## 2 Results and Discussion

### 2.1 Synthesis and Characterization of DEGTA

**Figure 2:**
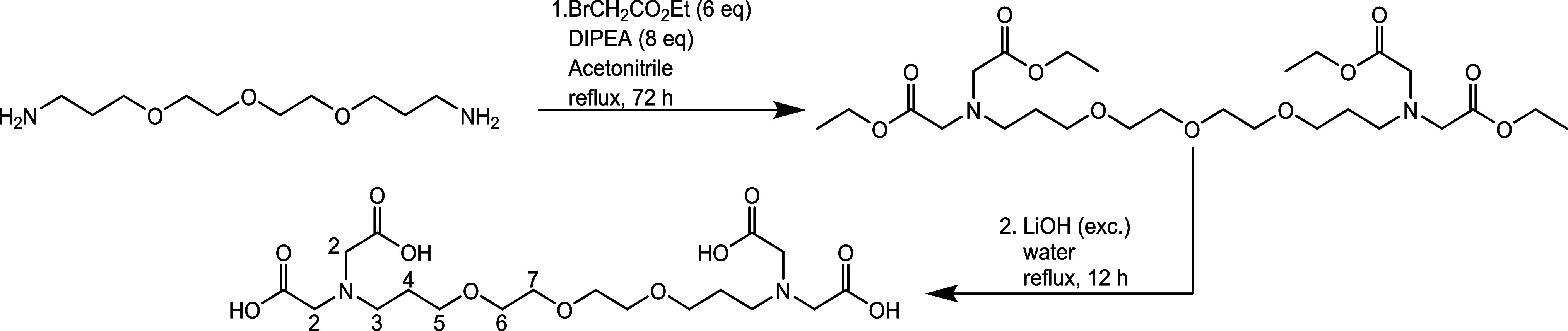
Synthesis route of the title compound along with labeling of the proton sites used for NMR signal assignment.

**Figure 3:**
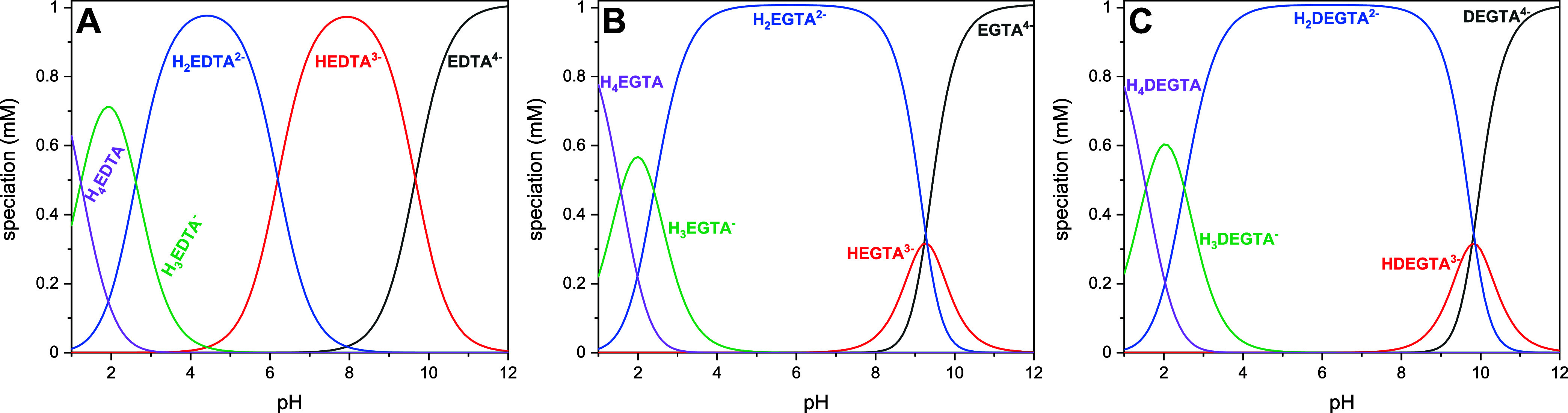
Calculated speciation of EDTA^1^ (A), EGTA^1^ (B), and DEGTA (C) using the p*K*_a_ values from Table 1. [Ligand] = 1 mM, *I* (NaCl) = 0.1 M, pH 1–12.

DEGTA was synthesized following established experimental procedures.^17,18^ This procedure allows for a one-pot synthesis for transforming the primary amino groups into tertiary amines. The complete addition of all four ethyl-protected carboxyl groups was tracked by using electrospray-ionization mass spectrometry (ESI-MS). In a second step, the acetate groups were deprotected to give the final product, with a yield of 34%. The successful ligand synthesis is confirmed by multinuclear NMR spectroscopy (Figure 2). Owing to exchange reactions (de/protonation equilibria) among coexisting species occur fast on the NMR time scale, the spectra represent mole fraction-weighted averages. Since these individual species give rise to individual chemical shifts, and also the signals’ relative order changes, we recorded full sets of H–C and H–N correlation spectra (provided in Figures S1–S4) at three different pD values. That is, pD 1.0, 6.0, and 12.0, for which H_4_L^0^, H_2_L^2–^, and L^4–^ are the respectively predominating species (Figure 3). Correspondingly, by symmetry, the ^1^H, ^13^C, and ^15^N spectra, respectively, exhibit always only six, seven, and one signal(s).

In general, the ^1^H signal associated with the aminoacetate methylene groups, 2, is the only one appearing as a singlet and is twice as intense as the others (in terms of its integral); cf. Figure 2 for H atom labeling and Figures S1–S4 for corresponding spectra. Assignment of the propyl residues is straightforward as the signals of their *N*-neighboring methylene groups (3) always resonate below 3.4 ppm and those of the *O*-neighboring CH_2_ groups (5) always resonate above 3.4 ppm. The signal arising from the CH_2_ group embedded by the former two (4) is the one most upfield and of highest multiplicity due to vicinal coupling to two adjacent CH_2_ groups (^3^*J*_H4,H3/5_). In contrast, signal assignment of the ethylene glycolic residues is not straightforward, requiring H–C correlation spectra. By means of better resolved ^13^C signals in combination with correlation experiments, assignment is unambiguous. The heteronuclear multiple bond correlation (HMBC) spectra consistently reveal correlation of sites 5 only to 6 as well as of 7 only to 6 (and to the magnetically nonequivalent site denoted 7’), but none between C5/H5 and C7/H7. Complementarily, nuclear Overhauser enhancement spectroscopy (NOESY) reveals only one nontrivial contact, viz. for H5 and H6, indicating those protons to be separated by the ether oxygen. Along with the single ^15^N NMR signal in the characteristic tertiary amine region shifting upfield with increasing pH, i.e., upon deprotonation, all NMR spectral features taken together prove the successful synthesis of the desired target molecule DEGTA.

#### 2.1.1 p*K*_a_ Determination

For further thermodynamic calculations, the ligand’s p*K*_a_ values have been determined by means of ^1^H NMR spectroscopy according to the literature^1^ (Figures S5–S7). Table 1 summarizes the determined p*K*_a_ values of DEGTA at a given ionic strength as well as ionic strength corrected using the specific-ion-interaction (SIT) theory. For comparison, the literature p*K*_a_ values of DEGTA’s smaller relatives, EDTA and EGTA, are also given.

**Table 1:** p*K*_a_ Values of DEGTA, Determined by NMR Spectroscopy.^a^

	p*K*_a_				
L	H_4_L^0^ ⇌ H_3_L^–^ + H^+^	H_3_L^–^ ⇌ H_2_L^2–^ + H^+^	H_2_L^2–^ ⇌ HL^3–^ + H^+^	HL^3–^ ⇌ L^4–^ + H^+^	
EDTA^1^	1.12 ± 0.06	2.50 ± 0.02	6.10 ± 0.01	9.65 ± 0.01	*I* = 0.1M NaCl
	1.36 ± 0.06	2.96 ± 0.02	6.74 ± 0.01	10.45 ± 0.01	*I* → 0
EGTA^1^	1.45 ± 0.04	2.28 ± 0.08	9.25 ± 0.01	9.25 ± 0.01	*I* = 0.1 M NaCl
	1.69 ± 0.04	2.74 ± 0.08	9.89 ± 0.01	10.05 ± 0.01	*I* → 0
DEGTA	1.43 ± 0.06	2.39 ± 0.11	9.82 ± 0.01	9.82 ± 0.01	*I* = 0.1 M (Li/Na)Cl
	1.67 ± 0.04	2.85 ± 0.07	10.46 ± 0.01	10.62 ± 0.01	*I* → 0

a pKa values of EDTA and EGTA are given for comparison. *I* extrapolated to zero ionic strength. The SIT ion interaction coefficients of EDTA (H_3_EDTA^–^: – 0.33 ± 0.14, H_2_EDTA^2–^: – 0.37 ± 0.14, HEDTA^3–^: – 0.10 ± 0.14, EDTA^4–^: 0.32 ± 0.14, all in NaCl^19^) were used as analogues for the calculations of DEGTA^4–^.

Calculated speciation of the ligand in comparison to EDTA and EGTA^1^ is given in Figure 3. This ligand speciation is valuable reference data to subsequently assess and evaluate the metal ion complexation studies. In a strongly acidic medium, DEGTA is present in its charge-neutral zwitterionic form (H_4_DEGTA^0^). Upon increasing the pH, one of the two protonated carboxyl groups deprotonates (H_3_DEGTA^–^). It is impossible to say which one as they are indistinguishable in aqueous solution. Subsequently, the remaining carboxyl group deprotonates as well (H_2_DEGTA^2–^). This species dominates over a wide pH range (pH 4–8). In alkaline media, the amine nitrogens deprotonate. This happens practically simultaneously; i.e., the two inflections are unresolved within the given pH-titration step size of ∼0.25 pH units. Applying a sigmoidal bidose–response fit, no second inflection point is found, in contrast to the carboxyl groups. DEGTA^4–^ is then present in a strongly alkaline environment. A similar behavior was observed for EGTA (Figure 3B). On the other hand, EDTA shows a different behavior, and both p*K*_a_ values from the amino groups can be observed independently (see Figure 3A). This is based on the shorter backbone where the two ammonium groups are not separated far enough, and they influence each other. In DEGTA (as well as in EGTA), the two ammonium groups are far enough separated from each other, so there is no Coulomb repulsion between them, which leads to the independent behavior and only two virtually identical p*K*_a_ values for both ammonium groups.

### 2.2 Complexation of DEGTA with Ln(III) and Cm(III)—Time-Resolved Laser-Induced Fluorescence Spectroscopy (TRLFS)

By taking advantage of the sensitive luminescence properties of the f-elements, spectroscopy is feasible at metal concentrations of less than 10^–5^ M. In this paper, TRLFS was used to determine the Ln(III)/An(III) DEGTA speciation, i.e., the number and thermodynamic stability of the forming complexes, also providing structural insights from the luminescing metal ion’s perspective.

In aqueous systems, de-excitation of the luminescing metal ion mainly facilitates via the use of O–H oscillators from coordinating water. Therefore, measurement of the luminescence lifetime τ enables approximation of the number of water molecules in the first coordination sphere, allowing one to conclude about the ligand coordination. For the Eu(III) ion, this was described by Horrocks et al. and yields eq 1.^20^ A similar relation was observed for Cm(III) by Kimura et al., corresponding to eq 2.^21^
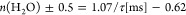
1
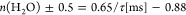
2

Furthermore, in the case of Eu(III), the number and splitting pattern of various ^5^D_0_ → ^7^F_J_ transitions are used to distinguish between different species and to gain insights into the complex symmetry.

**Figure 4:**
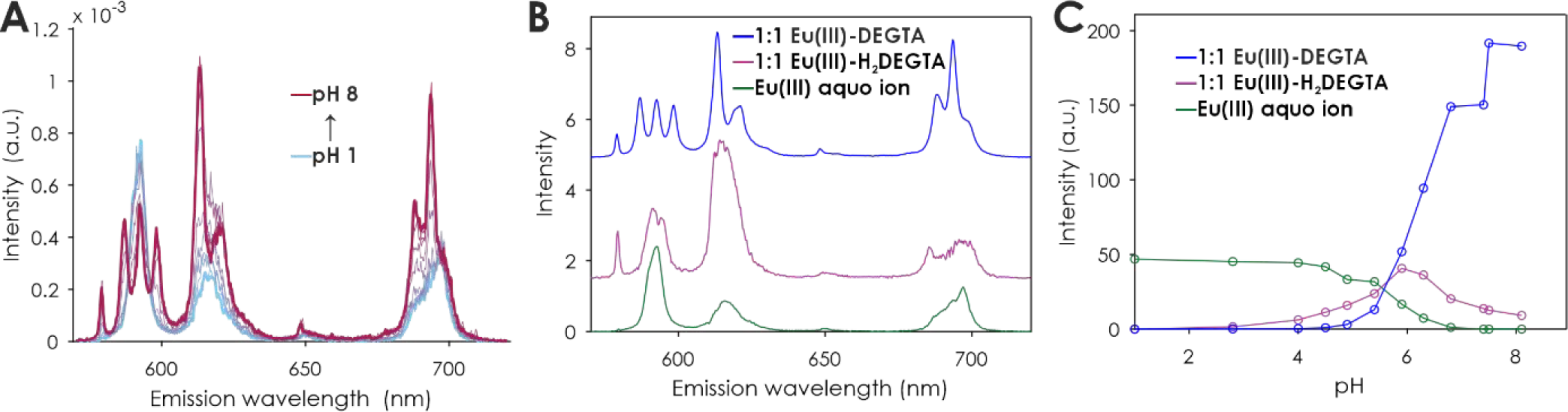
PARAFAC results of a pH-dependent TRLFS series of Eu(III) complexation by DEGTA. ^7^F_1_ normalized emission spectra at *t* = 0 μs (A), extracted single-component emission spectra (B), and luminescence intensity-based species distribution (C). [Eu(III)] = 10 μM, [DEGTA] = 100 μM, and *I* (Li/NaCl) = 0.1 M.

**Figure 5:**
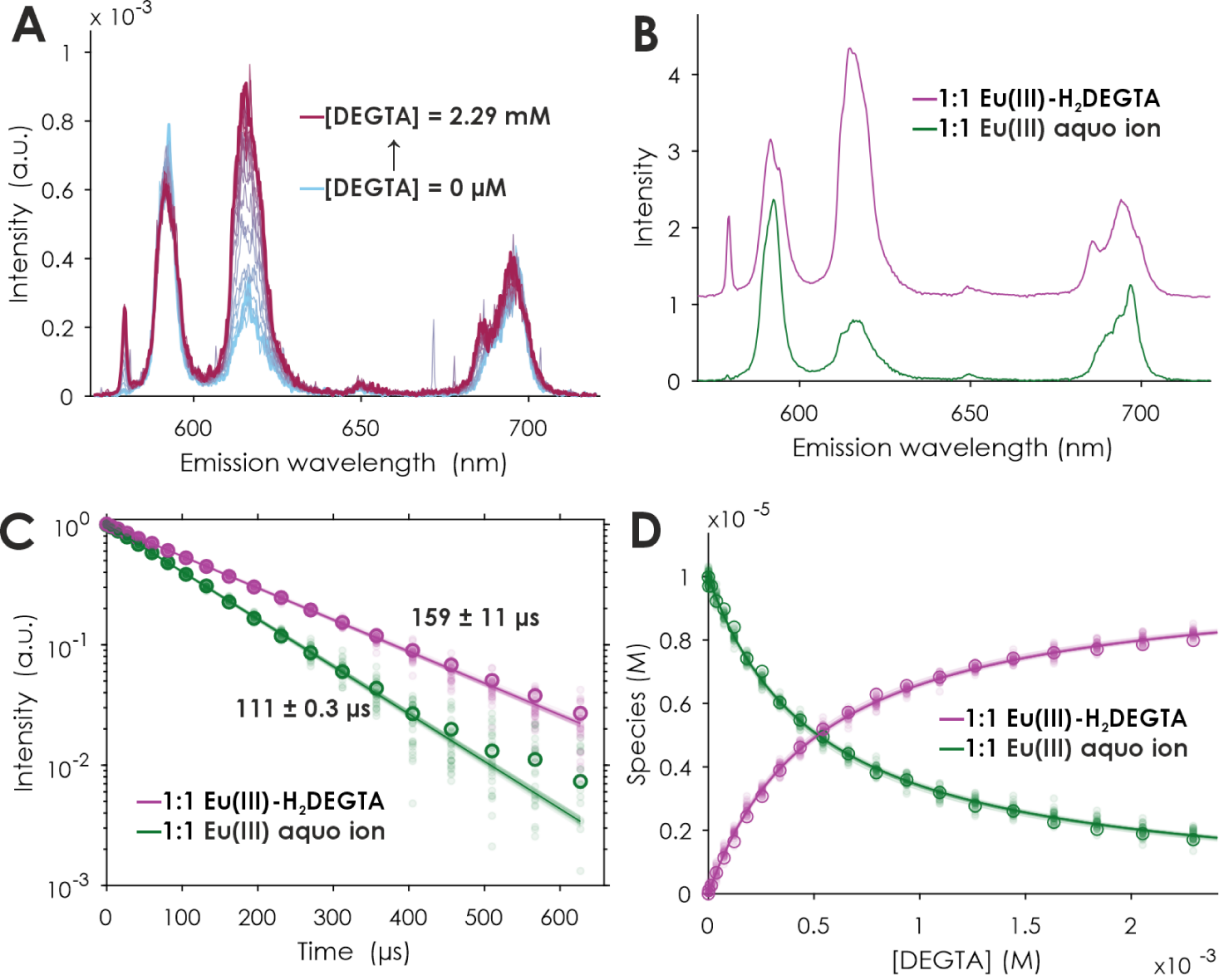
PARAFAC results of a TRLFS series of DEGTA complexation with Eu(III). ^7^F_1_ normalized emission spectra at *t* = 0 μs (A), extracted single-component emission spectra (B), luminescence decays (C), and quantum yield-corrected PARAFAC distributions (symbols) and corresponding speciation (lines) (D). [Eu(III)] = 10 μM, [DEGTA] = 0–2.29 mM, *I*(Li/NaCl) = 0.1 M, pH = 4.0 ± 0.1. The shaded data points were artificially created to be used in a Monte Carlo approach for the error estimation of the underlying model.

**Figure 6:**
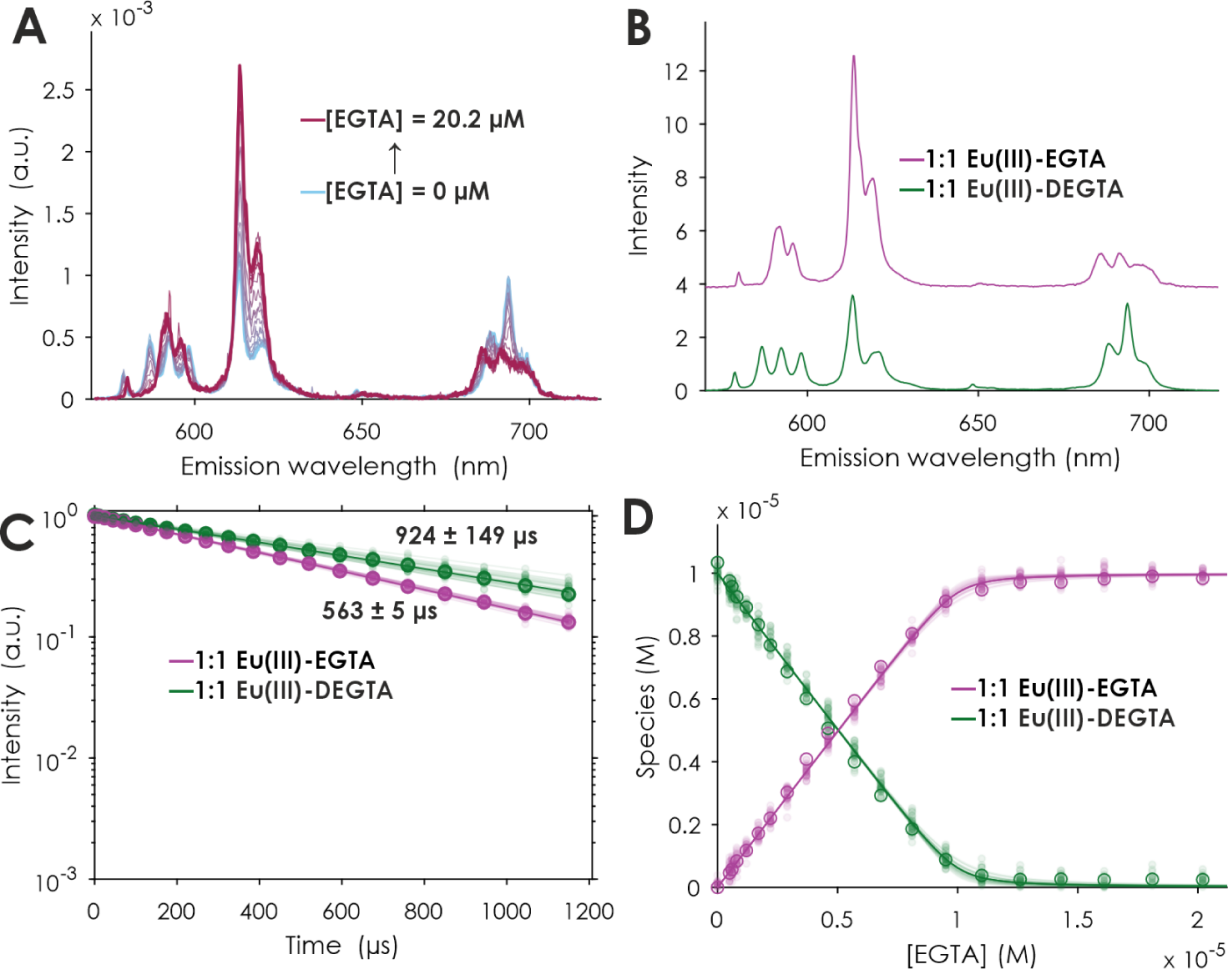
PARAFAC results of a TRLFS series of EGTA competition against DEGTA complexing Eu(III). ^7^F_1_ normalized emission spectra at *t* = 0 μs (A), extracted single-component emission spectra (B), luminescence decays (C), and quantum yield-corrected PARAFAC distributions (symbols) and corresponding speciation (lines) (D). [Eu(III)] = 10 μM, [DEGTA] = 20 μM, [EGTA] = 0–20.2 μM, [TRIS] = 1 mM, *I* (Li/NaCl) = 0.1 M, pH = 7.5 ± 0.1. The shaded data points were artificially created to be used in a Monte Carlo approach for the error estimation of the underlying model.

For complex systems, at difficult conditions, like varying pH or changing ligand concentrations, state-of-the-art mathematical tools are necessary to differentiate between multiple species, which partly occur simultaneously. Therefore, all TRLFS data were analyzed using parallel factor analysis (PARAFAC) as described elsewhere.^22^ PARAFAC is a generalization of PCA to higher order arrays. An advantage of applying PARAFAC to three-dimensional data (such as a set of TRLFS data) is that it overcomes the rotational issues inherent in bilinear two-dimensional methods thanks to its trilinearity. As a result, PARAFAC provides a unique and easy to interpret model.^23^ Briefly, using the simultaneous analysis of multiparameter data (pH- or concentration- or ligand-to-metal ratio-dependency of the emission spectra and luminescence decay lifetime), we determined a unique model with independent species. Three TRLFS series with Eu(III) and DEGTA were performed: (i) varying pH at given constant Eu(III) and DEGTA concentrations (Figure 4); (ii) titration of DEGTA to free Eu(III) at constant pH 4 (Figure 5); and (iii) titration of EGTA to [Eu(DEGTA)]^−^ at constant pH 7.5 (Figure 6).

TRLFS series with Eu(III) at varying pH showed that three different Eu species form. At low pH, the Eu(III) aquo ion is the only observable species (Figure 4C, green). Upon successive deprotonation of the ligand’s carboxyl groups, a second species occurs (Figure 4C, magenta). Based on the ligand’s p*K*_a_ values, at this pH the proposed species corresponds to [EuH_2_(DEGTA)]^+^. The extracted emission spectrum of this species (magenta lines in Figures 4B and 5B) shows a dominant ^5^D_0_ → ^7^F_0_ transition at 579.3 nm. This intense transition reflects a low coordination symmetry around the Eu(III) ion, implying that DEGTA binding is incomplete and supposedly “end-on”. This particular intense band along with the overall shape of the spectrum is comparable to the emission spectrum of the Eu(III)-NTA 1:1 complex, featuring a similar complex structure.^1^ That is, the single NTA ligand only partly covers Eu(III)’s coordination sphere, with the remaining sites saturated by (∼6) water molecules. Increasing the pH, the Eu(III) ion is able to replace remaining protons from the ligand and forms a third, distinct species (Figure 4C, blue). At the concentrations applied in TRLFS, this species starts forming at pH 5 and is the dominant species at pH > 6.5. Its luminescence intensity is much higher than those of the Eu(III) aquo ion and [EuH_2_(DEGTA)]^+^. The emission spectrum of this species (Figures 4B, blue, and 6B, green) includes a dominant ^5^D_0_ → ^7^F_1_ transition with a 3-fold splitting which is uncommon to observe at room temperature. Usually, this transition occurs as a single peak like in the Eu(III) aquo ion or the 1:1 complexes of europium with NTA or EDTA, or slightly 2-fold split like in the 1:1 complex with EGTA (Figure 6B, magenta).^1^ According to the multiplicity rule (2*J* + 1 with *J* = 1), this is the highest possible splitting for this transition,^24^ indicating a high degree of symmetry and embrace of the central ion by the ligand.

For a further description of the two formed DEGTA complex species and to obtain thermodynamic parameters, concentration-dependent titration series were conducted under conditions extracted from the pH-dependent series. The first series included a titration of DEGTA to free Eu(III) at pH 4.0 ± 0.1 (Figures 5 and S8). The lifetimes of the two observed species are 111 ± 0.3 and 159 ± 11 μs for the Eu(III) aquo ion and [EuH_2_(DEGTA)]^+^, respectively (Figure 5C, Table 2). According to eq 1, these lifetimes refer to 9.0 ± 0.5 and 6.1 ± 0.5 water molecules remaining in the coordination sphere of the Eu ion. Based on this and since the backbone is quite long and the amino groups remain protonated at this pH, only one side of the ligand seems to be bound, while the other side of the ligand is entropically disfavored to reach the Eu(III) ion. So in the [EuH_2_(DEGTA)]^+^ species, two carboxyl groups from one side of the ligand are bound to the Eu atom.

**Table 2:** Summarized TRLFS Data of Eu(III) and Cm(III) Complexes with DEGTA and Comparable Multidentate Ligands as well as the Free Aquo Ions

species	lifetime (μs)	n_H2O_ ± 0.5	^6^D_7/2_ → ^8^S_7/2_ peak maximum (nm)	ref.
[EuH_2_(DEGTA)]^+^	159 ± 11	6.1	-	this work
[Eu(DEGTA)]^−^	924 ± 149	0.5	-	this work
Eu(III)_aq_	110 ± 5	9.1		(1)
[Eu(EDTA)]^−^	299 ± 6	3.0	-	(1)
[Eu(EGTA)]^−^	586 ± 5	1.2	-	(1)
[Eu(DTPA)]^2–^	629	1.1	-	(27)
[Eu(3,4,3-LI(1,2-HOPO))]^–^	805 ± 81	0.7	-	(28)
[Cm(DEGTA)]^−^	448 ± 12	0.6	608.8	this work
Cm(III)_aq_	67 ± 5	8.8	593.8	(1)
[Cm(EDTA)]^−^	137 ± 5	3.9	603.7	(1)
[Cm(EGTA)]^−^	262 ± 5	1.6	609.1	(1)
[Cm(DTPA)]^2–^	268	1.5	606	(30)
[Cm(3,4,3-LI(1,2-HOPO))]^–^	383 ± 38	0.8	610	(31)

The stability constant of [EuH_2_(DEGTA)]^+^ was calculated as log *K* = 3.25 ± 0.10 (log β_112_ = 22.9 ± 0.1, according to β_MLH_ where *M*, *L*, and *H* denote the stoichiometric coefficients of metal ion, ligand, and protons involved, respectively) at a given ionic strength (0.1 M Li/NaCl) by PARAFAC,^25^ extrapolation to zero ionic strength using SIT yielded log *K*^0^ = 4.59 ± 0.10 (log β_112_^0^ = 25.7 ± 0.1), see Tables 3 and S1. This is in the range of the stability constants from comparable systems with a similar binding motif, such as the 1:1 complexes of Eu(III) with the dicarboxylic acids malonic acid, succinic acid, glutaric acid, and adipic acid. These complexes show stability constants of log *K* = 4.18 ± 0.01, 2.99 ± 0.01, 2.66 ± 0.01, and 2.59 ± 0.01 (*I* = 0.1 M), respectively.^26^ Surprisingly, although [EuH_2_(DEGTA)]^+^ structurally compares best with glutaric acid (both forming 8-membered chelate rings), the complex formation constant is over 1 order of magnitude higher (log *K* = 3.25 ± 0.1 and 2.66 ± 0.01, respectively).

To characterize the [Eu(DEGTA)]^−^ complex, which in the micromolar concentration range forms only at pH values close to commencing hydrolysis of the free Eu(III) aquo ion (see Figure 4C, blue), a competition reaction was used. Starting with the [Eu(DEGTA)]^−^ complex to circumvent the hydrolysis reaction, another ligand (EGTA) was added stepwise in order to displace DEGTA from the coordination sphere of Eu(III) (Figures 6 and S9). The kinetics of this displacement are surprisingly fast at room temperature, as seen by the instant detection of the Eu-EGTA complex-associated luminescence spectrum. Upon increasing the EGTA concentration in the solution, the spectrum of [Eu(DEGTA)]^−^ with the triple split ^5^D_0_ → ^7^F_1_ transition disappears, and the corresponding [Eu(EGTA)]^−^ with a slightly double split ^5^D_0_ → ^7^F_1_ transition appears (see Figure 6A). PARAFAC analysis yields two single-component spectra, and the calculated lifetimes of the observed species are 924 ± 149 and 563 ± 5 μs for the [Eu(DEGTA)]^−^ and [Eu(EGTA)]^−^ complex, respectively. The lifetime of [Eu(EGTA)]^−^ is in excellent agreement with previously published data.^1^ These lifetimes, respectively, calculate to 0.5 ± 0.5 and 1.3 ± 0.5 water molecules remaining in the coordination sphere of the Eu ion (Table 2).

The stability constant of [Eu(DEGTA)]^−^ was calculated as log *K* = log β_110_ = 16.3 ± 0.1 at a given ionic strength (0.1 M Li/NaCl) by PARAFAC, and extrapolation to zero ionic strength using SIT yielded log β_110_^0^ = 18.8 ± 0.1 (Table 3). Since this ligand is related to EDTA and EGTA, a direct comparison of the obtained figures is obvious. The increased lifetime of the [Eu(DEGTA)]^−^ species surpasses the lifetimes of Eu(III) complexes with other ligands providing eight coordination sites like the 1:1 complexes with EGTA, DTPA, and 3,4,3-LI(1,2-HOPO) (586 ± 5^1^, 629^27^, and 805 ± 81 μs,^28^ corresponding to 1.2 ± 0.5, 1.1 ± 0.5, and 0.7 ± 0.5 remaining water molecules, respectively, see Table 2). This indicates that the additional ether group in the backbone of the DEGTA is actually bound to the metal, totaling nine coordinating atoms (4 carboxyl, 3 ether oxygens, and 2 amino nitrogens). The stability constants of the 1:1 complexes of Eu(III) with EDTA, EGTA, and DEGTA, however, show no straightforward trend in this series (17.0 ± 0.1, 17.9 ± 0.1, and 16.3 ± 0.1 (0.1 M Li/NaCl), respectively, see Table 3). It seems as if the additional binding site in DEGTA, which is an ether group and therefore suspected to be weaker bound, cannot compensate for the increased backbone size as for EDTA vs EGTA (6 vs 8 binding sites, Figure 1). Therefore, this ligand appears to be sterically self-hindering and thus is too large for the Eu(III) ion. In addition, the higher p*K*_a_ values of DEGTA (Table 1) hinder Eu(III) ion proton displacement and thus lower the affinity.

**Figure 7:**
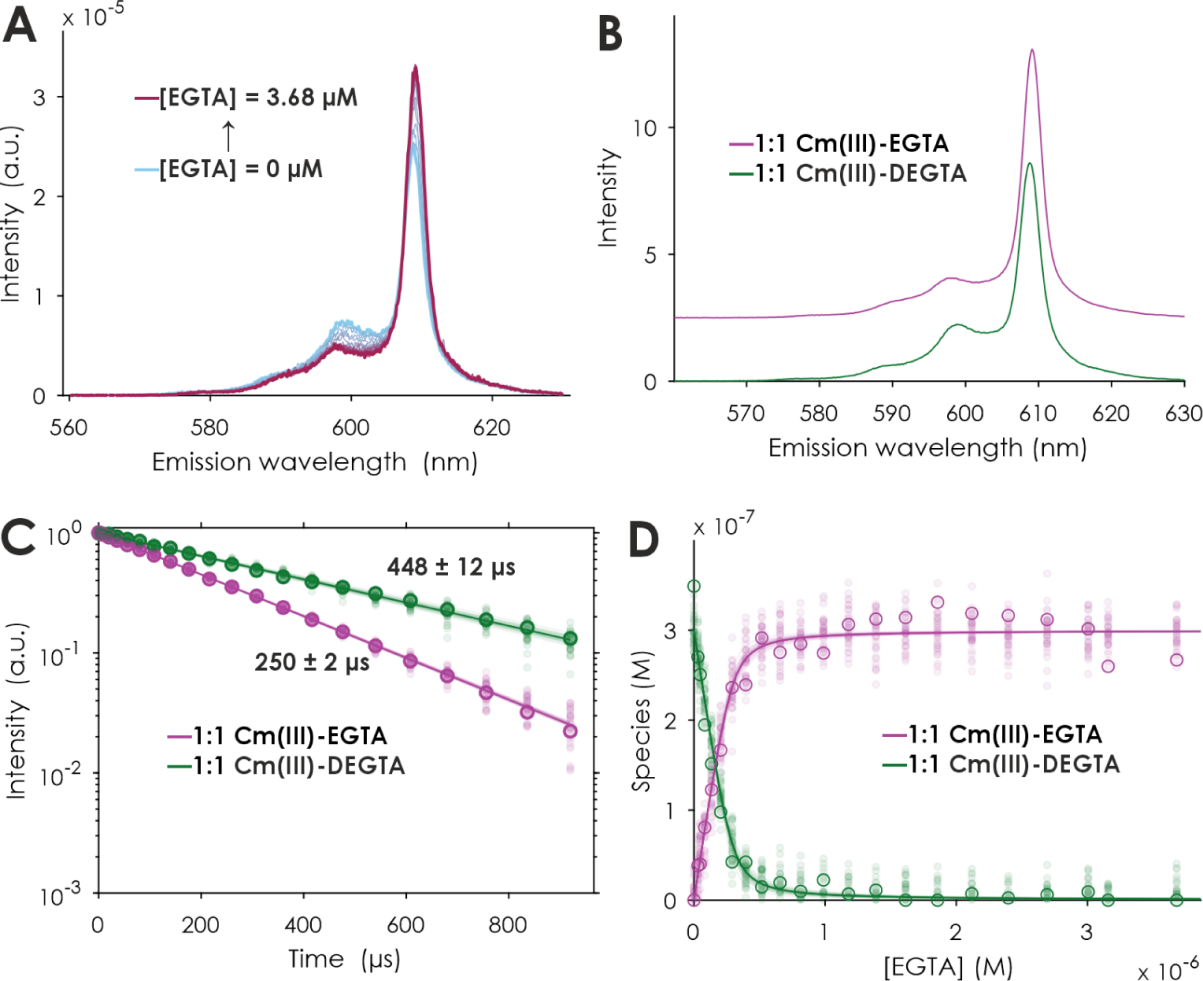
PARAFAC results of a TRLFS series of EGTA competitions against DEGTA complexing Cm(III). Normalized emission spectra at *t* = 0 μs (A), extracted single-component emission spectra (B), luminescence decays (C), and quantum yield-corrected PARAFAC distributions (symbols) and corresponding speciation (lines) (D). [Cm(III)] = 0.3 μM, [DEGTA] = 1 μM, [EGTA] = 0–3.68 μM, [TRIS] = 1 mM, *I* (Li/NaCl) = 0.1 M, pH = 7.5 ± 0.1. The shaded data points were artificially created to be used in a Monte Carlo approach for the error estimation of the underlying model.

To analyze Cm(III) complexation by DEGTA, the same competition approach was used (Figure 7). Upon an increase in the EGTA concentration, the emission spectrum reveals only minimal changes (Figure 7A). Nevertheless, PARAFAC analysis yields two single-component spectra with quite similar shapes (Figure 7B). Both spectra show blueshifted hot bands at around 598 nm and another smaller one at 589 nm.^29^ Cm(III) complexes of DEGTA and EGTA respectively show emission maxima at 608.8 and 609.1 nm, and the calculated lifetimes of 448 ± 12 and 250 ± 2 μs correspond to 0.6 ± 0.5 and 1.7 ± 0.5 water molecules remaining in the metal’s coordination sphere, according to eq 2 (Table 2). Like the Eu(III) system, the lifetime of [Cm(DEGTA)]^−^ exceeds those of the 1:1 Cm(III) complexes with octadentate ligands EGTA, DTPA, and 3,4,3-LI(1,2-HOPO) (262 ± 5^1^, 268,^30^ and 383 ± 38 μs,^31^ corresponding to 1.6 ± 0.5, 1.5 ± 0.5, and 0.8 ± 0.5 remaining water molecules, respectively, see Table 2). This further increased lifetime strongly implies that all nine coordination sites are bound to the Cm(III) ion, and no water is present in its first coordination sphere.

The stability constant of [Cm(DEGTA)]^−^ was calculated as log *K* = log β_110_ = 17.9 ± 0.1 at a given ionic strength (0.1 M Li/NaCl) by PARAFAC, extrapolation to zero ionic strength using SIT yielded log β_110_^0^ = 20.5 ± 0.1. This value is larger than that of the comparable Eu(III) system (Table 3). This trend of higher log β values for Cm(III) than for Eu(III) while using the same binding partner was previously reported in the literature for both EDTA and EGTA. In contrast to the Eu(III) system, [Cm(DEGTA)]^−^ does not show the smallest stability constant in the series of EDTA, EGTA, and DEGTA (17.5 ± 0.1, 18.6 ± 0.1, and 17.9 ± 0.1 (0.1 M Li/NaCl), respectively, see Table 3).^1^ Compared to EDTA and EGTA, in DEGTA either three or one additional ether coordination site is provided, respectively. These ether groups show less ionic and more covalent bonding behavior than carboxyl groups. Since actinides tend to form more covalent bonds,^32^ the additional ether group now compensates for the larger size of the backbone and instead adds some stability in the curium complex compared to the europium complex. Another noteworthy aspect is the quite similar shapes of the emission spectra of both [Cm(DEGTA)] ^–^ and [Cm(EGTA)]^−^. This may be based on the electronic nature of curium which, in contrast to europium, shows only one transition (^6^D_7/2_ → ^8^S_7/2_) that is excited in the TRLFS. Since the coordination sites of EGTA and DEGTA differ only by one additional ether oxygen in the latter, the ligand field is comparable. For the electronic transition in Cm(III), it seems that this small difference in the ligand field has nearly no influence on the emission spectrum. This difference in the ligand field is, for example, much larger when comparing the emission spectra of [Cm(EDTA)]^−^ and [Cm(EGTA)]^−^, corresponding to 6- and 8-fold coordination, respectively.^1^ Comparing, however, EGTA’s and DEGTA’s denticities of 8 and 9, respectively, the 8-fold coordination already approaches Cm(III)’s maximum coordination number so that increasing the number by one more coordinating atom the impact on the spectra is anticipated to be rather small.

### 2.3 Nuclear Magnetic Resonance Spectroscopy (NMR)

#### 2.3.1 Structure Determination

The aqueous solution complex structure was comprehensively studied from the ligand’s perspective by means of NMR spectroscopy, using DEGTA complexes of Eu(III) and Sm(III). Both constitute chemically closely related neighboring lanthanide ions, which, in this case, form isostructural complexes. However, due to Sm(III)’s 4f^5^ and Eu(III)’s 4f^6^ electron configurations, both exhibit distinct qualitative and quantitative paramagnetic effects in the NMR spectra, with those caused by Eu(III) being significantly larger. They include both paramagnetically induced shifts and enhanced relaxation, the latter translating into broadened lines up to collapsing the signals’ fine structure (splitting), while they are largely preserved in the ^1^H spectra of the Sm(III) complex. In both cases, acquisition of correlation spectroscopy (COSY), heteronuclear single-quantum coherence (HSQC), and NOESY spectra was feasible, allowing for correlation of ^1^H and ^13^C signals and eventually unambiguously assigning all signals and elucidating the molecular structure of the complexes.

**Figure 8:**
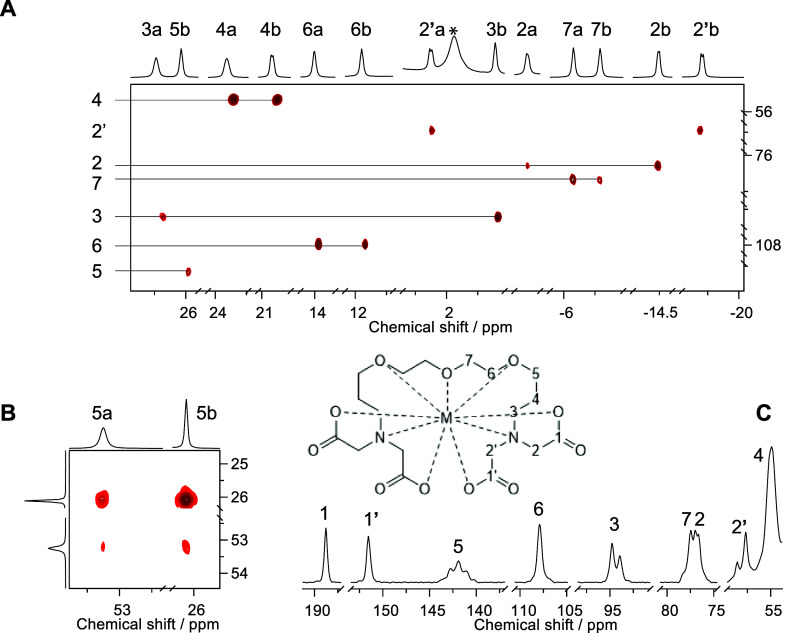
Representative NMR spectra of the Eu(III)–DEGTA complex in D_2_O solution containing 20 mM Eu(III) and excess ligand at pD 8 along with signal assignment corresponding to labeling given with the structure (insert). (A) H,C-HSQC spectrum depicting connectivity of diastereotopic hydrogens and the carbons they are attached to. (B) Magnification of the COSY spectral region showing the correlation between hydrogen atoms 5a and 5b, complementing the one CH correlation not observed in (A). (C) ^13^C{^1^H} spectrum (lb = 50 Hz) showing complex-associated signals. For the sake of clarity, only regions of interest are presented. The asterisk indicates a signal due to an excess ligand.

Figure 8 depicts spectra associated with the [Eu(DEGTA)]^−^ complex and the corresponding signal assignment. Additional spectra of both Sm and Eu complexes along with a table summarizing observed ^1^H and ^13^C chemical shift values of the free ligand at three different pH values (cf. section p*K*_a_ determination) juxtaposed to those of the Sm and Eu complexes are provided as Supporting Information (Table S2 and Figures S10–S17).

As can be seen in Figure 8, complexation of Eu(III) causes ^1^H signals to shift down to 53.2 ppm (H5a) and up to −19.4 ppm (H2’). Such extreme shifts result in incomplete ^1^H broadband decoupling (almost 30 kHz width), causing the corresponding ^13^C signals to show some residual coupling, as is the case especially for 5, 3, 2, and 2’. Carbons either bearing protons resonating in a moderate chemical shift range (such as 4, 6, and 7) or being quaternary such as those of the carboxyl groups (1 and 1’) appear as singlets. In addition to the drastic shift, signal 5a is also remarkably broadened, and hence, no C5–H5a correlation is observable. However, unambiguous assignment of the signal at 53.2 ppm is achieved via the geminal scalar coupling (^2^*J*_5a,b_) correlation observable in the COSY. A full depiction of the spectrum in Figure S12 shows the numerous additional *vicinal* (^3^*J*) and ^2^*J* couplings facilitating assignment upon identification of (isolated) spin systems such as the propyl residue (3a/b, 4a/b, 5a/b), the ethylene glycol residue (6a/b, 7a/b), and the acetate residues (2 a and b as well as 2’ a and b). A ^4^*J* between protons of nonequivalent acetate residues is not observed. However, both a weak scalar ^4^*J*_2’b,3b_ and dipolar couplings (NOE contacts) between 3(a/b) and 2/2’(a/b) are detectable. Further spatial relationships inferred from the NOESY are in line with the DFT-calculated structure.

The DEGTA complexes of both Sm(III) and Eu(III) are isostructural and possess *C*_2_ symmetry since the aminoacetate residues are pairwise equivalent by symmetry (cf. Figures 10, S28 and S29). Accordingly, the carboxyl carbons (1 and 1’) as well as the carbons and protons of the adjacent methylene groups (2 and 2’) respectively show distinct signals of equal intensity. In addition, all methylene groups are diastereotopic, and thus, their protons give rise to individual signals (denoted a/b in Figure 8) in some cases of remarkable frequency differences. This is caused by the hyperfine interaction, i.e., (mainly) by distance- and angle-dependent pseudocontact (especially ^1^H) and to some extent maybe also by Fermi-contact contributions. The calculated pseudocontact field isosurface plot (Figure S29) is in qualitative agreement with the complex’ geometry and, correspondingly, the sign (i.e., shielding vs deshielding effects) and magnitude of the paramagnetic shifts observed in the NMR spectra.

Eventually, the COSY and HSQC spectra provide clues on atom connectivity. Furthermore, NMR evidence shows that the ligand binds the metals Eu(III) and Sm(III) by all nine coordination sites. Differences in the magnitude of the respective (induced) chemical shifts are by no means indicators of strong and weak binding sites; the more they mirror the effects of magnetic anisotropy caused by the metal ions’ f electrons. In the case of the diamagnetic La(III) containing sample, the complex appears to be significantly different regarding its low kinetic stability, as concluded from the weak shifts and severe line broadening (cf. Figure S23). Even at high overall concentrations and only slight ligand excess, the spectrum of the La(III) containing sample resembles that of the free ligand but is notably broadened. In the presence of Eu(III) and still a 2-fold excess of La(III), signals characteristic of [Eu(DEGTA)]^−^ appear (middle and bottom spectrum in Figure S23A) indicative of the significantly higher kinetic (and likely also thermodynamic) stability of the Eu(III) DEGTA complex compared to that of La(III). These indications are reflected by significantly larger bond lengths between La(III) and all coordinating atoms compared to Eu(III), cf. Figure S25 and Table S3.

#### 2.3.2 Concentration- and pD-Dependent NMR-Titration Series of DEGTA with Eu(III)

Concentration- and pD-dependent solutions were examined by NMR (Figures S18–S23) to corroborate the first Eu(III) complex species, [EuH_2_(DEGTA)]^+^, deduced from TRLFS. These series revealed the following major results. Metal ion concentrations in the millimolar range (1.0–50 mM) as used for NMR measurements cause the formation of the nine-coordinate complex to commence already at remarkably lower pH compared to the μM TRLFS conditions, e.g., at 50 mM Eu and 2 mM ligand at pD as low as 3.1 (Figure S18). This is due to the outcompetition of H^+^ by Eu^3+^, in this case at a Eu^3+^/H^+^ ratio of about 60, consequently effectively displacing the ammonium hydrogen atoms, yielding the complete nine-coordinate complex. The latter is by far the most prominent species easily recognized by its characteristic signals (cf. Figure 8 as well as Figures S18–S23). A further set of signals distinct from the former and similar but different from those of the free ligand are detectable in the range 3 ≤ pD < 6 and predominating around pD 4 at NMR concentrations. The observation under sufficiently acidic conditions along with the spectral appearance is in well accordance with “end-on” coordination to the metal ion by one pair of carboxyl groups as suspected for [EuH_2_(DEGTA)]^+^ from TRLFS. Taking into account the weak stability of that complex (log *K* of 3.25 ± 0.1) and the fact that the observed signals are mole fraction-weighted averages due to rapid exchange between free and rather weak end-on binding ligand, spectra resemble those of the free ligand especially for excess ligand. However, especially for excess metal ions and sufficiently low pD to prevent the complete chelation, spectra display more end-on complex character. Correspondingly, the pD 4.1 and 5.1 spectra in Figure S19 display broad signals more or less displaced relative to those of the blank. Of these, the largest are observed for the aminoacetate methylene signals (highlighted in Figures S19B and S20B) and attenuating along the molecular chain with effects successively decreasing along the propyl residue, in line with the end-on binding and the residual sites in the molecule pointing away from the paramagnetic metal ion. Further increasing pD via 5 to 6 causes the [EuH_2_(DEGTA)]^+^ species to be replaced by [Eu(DEGTA)]^−^ as represented by the increasing intensity (integral) of the corresponding signal 2’a.

### 2.4 Fourier-Transform Infrared (FT-IR) Spectroscopy

**Figure 9:**
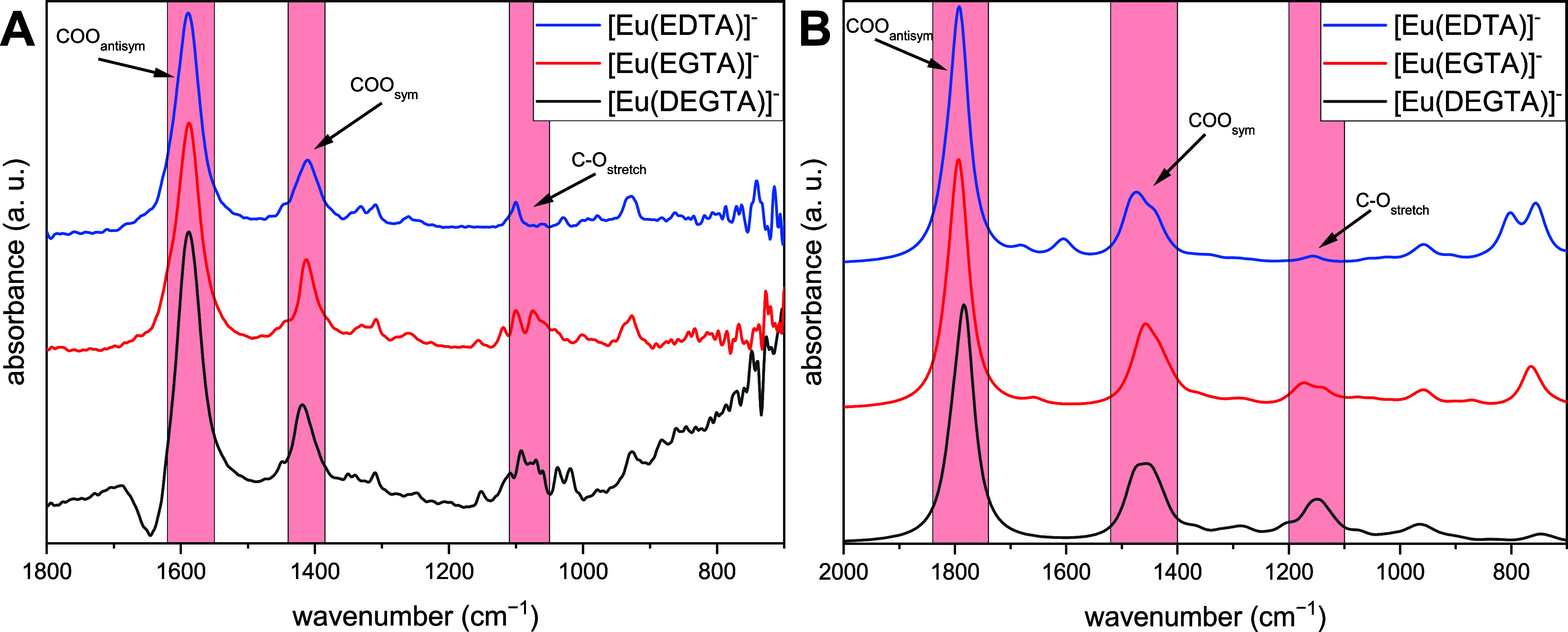
Measured (A) and calculated (B) FT-IR spectra of the 1:1 complexes of Eu(III) with EDTA, EGTA, and DEGTA. [Eu(III)] = [ligand] = 10 mM, *I* (NaCl) = 195 mM, pH = 9.

Comparing the FT-IR measured spectra of [Eu(DEGTA)]^−^ and related complexes [Eu(EDTA)]^−^ and [Eu(EGTA)]^−^ (Figure 9A), virtually identical band positions evidence a similar binding motif. That is, in all three spectra bands due to COO antisymmetric and symmetric stretching vibrations are observed at 1590 and 1410 cm^–1^. Along with the fact that in single-crystal X-ray structures the carboxyl groups in [Eu(EDTA)]^−^ and [Eu(EGTA)]^−^ feature monodentate coordination,^34,35^ one can conclude that this holds true also in [Eu(DEGTA)]^−^. As expected from the structures of the ligands (Figure 1), in the FT-IR-spectra of [Eu(EGTA)]^−^ and [Eu(DEGTA)]^−^, at around 1080 cm^–1^ the C–O stretching vibrations associated with ether residues are observable while they are absent in the spectrum of [Eu(EDTA)]^−^. The weak signal visible in this area of the [Eu(EDTA)]^−^ spectrum originates from the C–N stretching vibration of the amino residue, present in all spectra but overlapping with the aforementioned C–O stretch in both other spectra. These trends were confirmed by simulation of the corresponding IR spectra (Figures 9B, S26 and S27).

### 2.5 Density Functional Theory (DFT)

**Figure 10:**
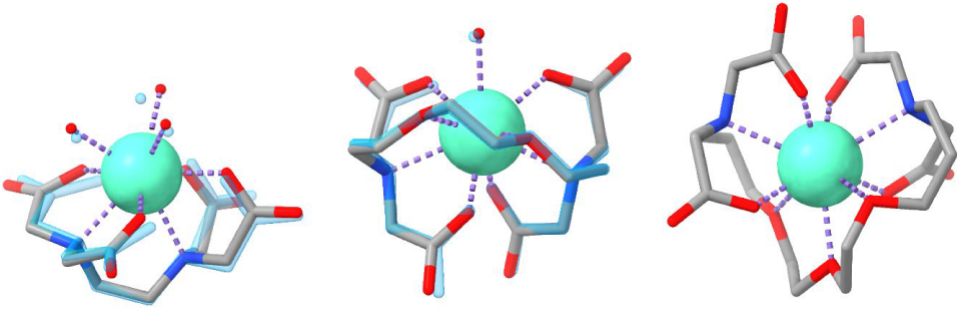
DFT-calculated structures of Eu(III) 1:1 complexes with EDTA (left), EGTA (middle), and DEGTA (right). Color code: green: Eu(III), red: oxygen, blue: nitrogen, gray: carbon. Protons are omitted. For the EDTA and EGTA complexes, respective crystal structures were overlaid in transparent light blue. For the larger sphere representation of Eu, the effective ionic radius for a 9-fold coordination by Shannon was chosen.^40^

The structures of the 1:1 complexes of Eu(III) and the three ligands EDTA, EGTA, and DEGTA were calculated in aqueous solution using density functional theory (DFT) (Figure 10) as well as the structures of the 1:1 complexes of La(III) and Cm(III) with DEGTA (Figure S25). The obtained geometries were furthermore used to perform more sophisticated single-point all-electron DFT calculations (see Computational Details), in order to obtain reliable electron density results later on used for quantum atoms in molecules (QTAIM) analyses^36,37^ yielding delocalization indices (DI) as a measure for the covalency of the ligand–metal bonds. The distances between Eu(III) and important (ligating) atoms are listed in Table 4 (for La(III) and Cm(III) data, see Table S3). Calculated DI values of all ligand–metal bonds for the investigated complexes are also listed in Table 4 and additionally shown in Figure S24 and listed in Table S3. For EDTA and EGTA, the calculated structures compare very well with known crystal structure data, which proves the robustness of the method and allows to draw direct conclusions and comparison.^34,35,38,39^ Minor deviations can be attributed to differences in macroscopic effects and long-range ordering within the solid structure and solution, both of which are strongly influenced by hydrogen bonding to and among water molecules present in these two cases.

**Table 3:** Formation Constants of Eu(III) and Cm(III) Complexes with EDTA, EGTA, and DEGTA, Determined by TRLFS

species	M L H	log β_MLH_	log β_MLH_^0^^b^
Eu^3+^ + DEGTA^4–^ + 2 H^+^ ⇌ [EuH_2_(DEGTA)]^+^	1 1 2	22.9 ± 0.1^a^	25.7 ± 0.1
Eu^3+^ + EDTA^4–^ ⇌ [Eu(EDTA)]^−^	1 1 0	17.0 ± 0.1^1^	19.5 ± 0.1^1^
Eu^3+^ + EGTA^4–^ ⇌ [Eu(EGTA)]^−^	1 1 0	17.9 ± 0.1^1^	20.5 ± 0.1^1^
Eu^3+^ + DEGTA^4–^ ⇌ [Eu(DEGTA)]^−^	1 1 0	16.3 ± 0.1^a^	18.8 ± 0.1
Cm^3+^ + EDTA^4–^ ⇌ [Cm(EDTA)]^−^	1 1 0	17.5 ± 0.1^1^	20.0 ± 0.1^1^
Cm^3+^ + EGTA^4–^ ⇌ [Cm(EGTA)]^−^	1 1 0	18.6 ± 0.1^1^	21.2 ± 0.1^1^
Cm^3+^ + DEGTA^4–^ ⇌ [Cm(DEGTA)]^−^	1 1 0	17.9 ± 0.1^c^	20.5 ± 0.1^c^

a *I* = 0.1 M (Li/NaCl). Errors are standard deviation of at least three independent experiments.

b *I* extrapolated to zero ionic strength. The SIT ion interaction coefficients used for the calculations were 0.26 ± 0.01 for Eu(III) in NaCl.^33^ For Cm(III), no data were available, and Am(III) coefficients (Am(III) in NaCl: 0.23 ± 0.02^19^ were used as substitute. For H_2_DEGTA^–^ and DEGTA^4–^, the data for EDTA (H_3_EDTA^–^: – 0.33 ± 0.14, H_2_EDTA^2–^: – 0.37 ± 0.14, HEDTA^3–^: – 0.10 ± 0.14, EDTA^4–^: 0.32 ± 0.14, all in NaCl^19^) were used.

c The given error is the fitting error of a single measurement.

**Table 4:** Selected Distances (in Ångström) and Delocalization Indices (DI) between Atoms from the Eu(III) 1:1-Complexes with EDTA, EGTA, and DEGTA^a^

	[Eu(EDTA)(H_2_O)_3_]^−^			[Eu(EGTA)(H_2_O)]^−^			[Eu(DEGTA)]^−^	
	calculated	measured^34^	DI	calculated	measured^35^	DI	calculated	DI
Eu–O_carb_	2.338	2.383(2)	0.32	2.313	2.374(2)	0.34	2.321	0.33
Eu–O_carb_	2.350	2.392(2)	0.30	2.339	2.386(2)	0.32	2.346	0.33
Eu–O_carb_	2.379	2.426(2)	0.30	2.346	2.389(2)	0.30	2.348	0.32
Eu–O_carb_	2.385	2.458(2)	0.28	2.382	2.394(2)	0.29	2.349	0.30
Eu–O_ether_	-	-		2.522	2.487(2)	0.17	2.516	0.17
Eu–O_ether_	-	-		2.542	2.540(2)	0.17	2.565	0.17
Eu–O_ether_	-	-		-	-		2.599	0.13
Eu–N	2.620	2.622(2)	0.20	2.622	2.637(3)	0.20	2.748	0.15
Eu–N	2.663	2.727(2)	0.18	2.668	2.661(3)	0.17	2.777	0.15

a O_carb_: carboxyl group oxygen. O_ether_: ether oxygen from backbone.

The calculated structure of the Eu(III)-DEGTA 1:1 complex is in good agreement with NMR results (Figure 8), proving that all nine heteroatoms from the DEGTA molecule (4 carboxyl and 3 ether oxygen atoms, and 2 amino nitrogen atoms) are actually bound to the Eu(III) ion as expected from the structure of the ligand (Figure 1) and the TRLFS lifetime results (Figure 6C). The structure also compares well with the calculated structures of the 1:1 complexes of Eu(III) with EDTA and EGTA. All four carboxyl groups are monodentately bound (κ^1^) to the metal center as experimentally observed by FT-IR (Figure 9A) and confirmed by simulation (Figures 9B, S26 and S27). Conformer search calculations via xTB/CREST^41,42^ (see Computational Details) have suggested the existence of many (initially) energetically low-lying conformers with a more open structure of the backbone. In these cases, most of the time, only one or two ether oxygen atoms coordinate, while at least two of the carboxyl groups seem to become bidentate (κ^2^). The nitrogen atoms are basically nonbound. DFT optimization (with implicit water solvation) of the energetically lowest conformer found within these structures, however, showed a destabilization by 103 kJ/mol in the Gibb’s free energy (Figure S28), which translates to a Boltzmann population of around 10^–16^, rendering this form irrelevant at ambient conditions.

The mean distances between the binding carboxyl group oxygen and the europium ion in [Eu(EDTA)(H_2_O)_3_]^−^, [Eu(EGTA)(H_2_O)]^−^, and [Eu(DEGTA)]^−^ are 2.36 ± 0.02, 2.35 ± 0.02, and 2.34 ± 0.01 Å, respectively. This shows that, regardless of the size of the backbone, the carboxyl groups bind with the same strength, also seen in the almost similar DI values of the QTAIM analysis (0.30 ± 0.02, 0.31 ± 0.02, 0.32 ± 0.02, respectively). The backbone ether oxygen atoms are also quite similarly bound (2.53 ± 0.01 and 2.56 ± 0.03 Å for [Eu(EGTA)(H_2_O)]^−^ and [Eu(DEGTA)]^−^, respectively). These bond lengths are associated with the nature of the functional group, which is somewhat weaker bonded than the carboxyl groups (DI_EGTA_(O_carb_–Eu) = 0.31, DI_EGTA_(O_ether_–Eu) = 0.17) and therefore longer but still almost independent of the length of the backbone. An exception is presented by the central ether oxygen in the DEGTA complex which, although only slightly farther away from Eu than the other two O_ether_ atoms, exhibits a noticeably smaller DI (0.13 vs 0.17). This was also calculated for the corresponding Cm(III) complex (0.16 vs 0.20 and 0.21, respectively). The most prominent difference is observable for the distances between the Eu(III) ion and amino group nitrogen atoms: their mean bond lengths are 2.64 ± 0.02, 2.65 ± 0.02, and 2.76 ± 0.01 Å in [Eu(EDTA)(H_2_O)_3_]^−^, [Eu(EGTA)(H_2_O)]^−^, and [Eu(DEGTA)]^−^, respectively. The larger Eu–N distance in the DEGTA complex might stem from steric hindrances and strains that come with the elongated backbone of the DEGTA ligand compared to EDTA and EGTA: the amino groups are thus prevented from getting closer to the metal center. Comparing the DI values of [Eu(DEGTA)]^−^ and [Cm(DEGTA)]^−^ (Table S3), it is obvious that the DI values in the Cm(III) complex are slightly higher in general. This addresses the increased covalency in the binding motif of the actinide as discussed earlier and in literature.^32^

**Figure 11:**
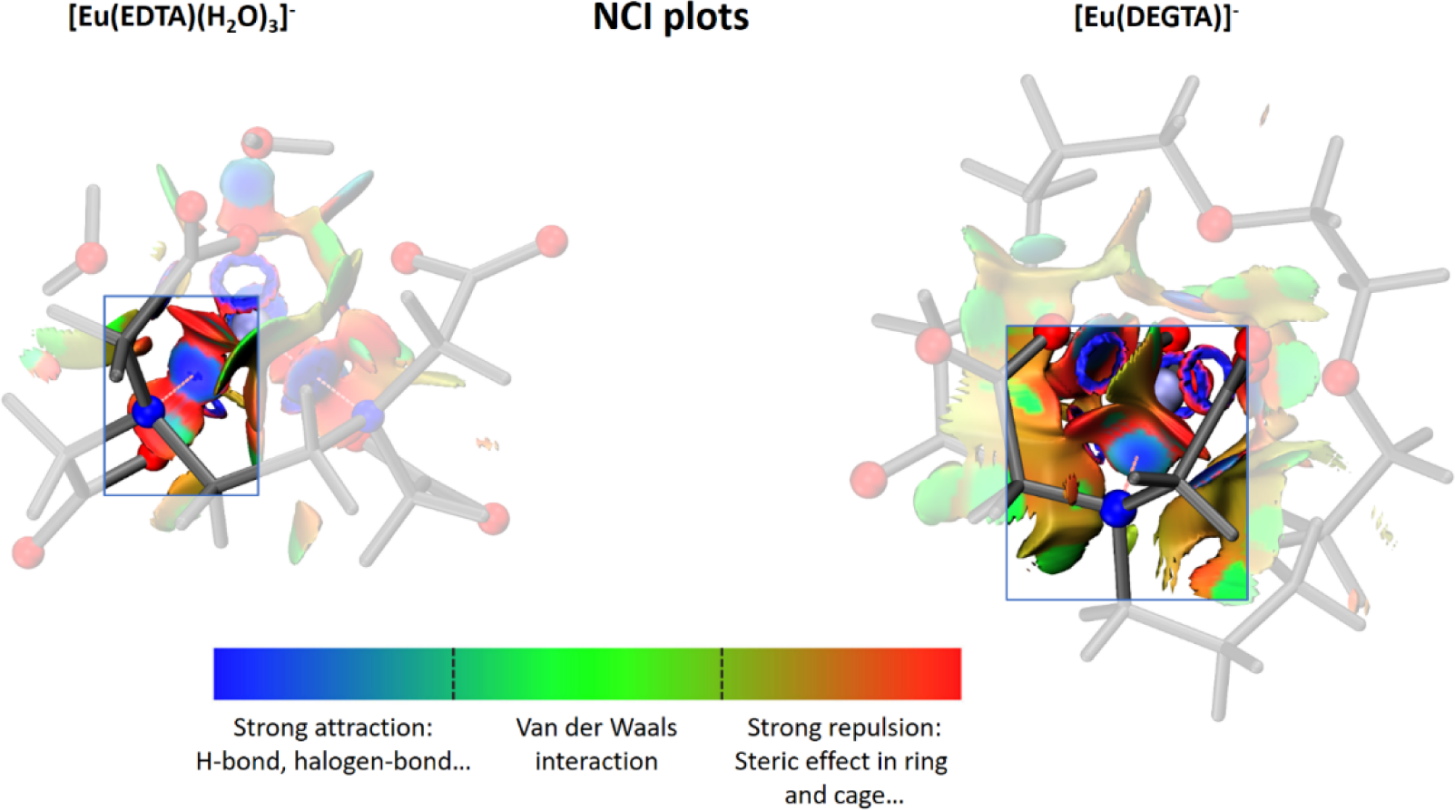
NCI plots of [Eu(EDTA)(H_2_O)_3_]^−^ (left) and [Eu(DEGTA)]^−^ (right). The color code was taken from the Multiwfn manual.^43−45^

These steric aspects and suspected weaker Eu(III)–N interactions were confirmed by noncovalent interaction (NCI) plots (Figure 11) derived from initial ECP-based DFT calculations using Multiwfn.^45^ For [Eu(EDTA)(H_2_O)_3_]^−^, they reveal a strong attraction of N to the central Eu and the only repulsive areas are marked by neighboring O_carb_–Eu bond densities. By contrast, in [Eu(DEGTA)]^−^ the environment in the direct vicinity of the Eu–N bond is dominated by repulsive forces originating from steric effects of the backbone, while the attractive area between N and Eu already declines within the color scale, indicating a weaker attraction. This weaker Eu(III)–N bond also may be the main reason why the 1:1 complex of Eu(III) with the fully deprotonated DEGTA occurs only above pH 6.5, while the comparable EDTA and EGTA complexes occur at pH values as low as 2.0 and 2.5, respectively.^1^ The comparatively weaker bond and therefore longer distance between europium and nitrogen in [Eu(DEGTA)]^−^ make it more difficult for the metal to displace the protons in the ammonium form. Therefore, the final yield of the amine form, leading to the formation of the fully deprotonated complex with the Eu(III) ion, is only possible if the proton concentration in the surrounding solution is several orders of magnitude lower, and therefore less competition occurs between H^+^ and Eu^3+^. Additionally, this weaker bound coordination site makes it easier for competing ligands to attack the metal center. This behavior was proven and exploited in the titration of the competing ligand EGTA to a solution of [Eu(DEGTA)]^−^ (Figure 6) and [Cm(DEGTA)]^−^ (Figure 7).

## 3 Conclusion

To extend the family of aminopolycarboxylates, the new member DEGTA with an elongated backbone was successfully synthesized in a two-step synthesis. Both the ligand’s and the complex’s structures, as well as the (thermodynamic) complexing behavior toward Eu(III), Sm(III), La(III), and Cm(III) were comprehensively investigated using NMR spectroscopy, TRLFS, and DFT.

DEGTA forms two Eu(III) 1:1 complex species predominating depending on the pH. The first complex species occurring at pH values around 4 (in both TRLFS and NMR) shows a short luminescence lifetime (159 ± 11 μs) and a low complex stability constant (log *K* = 3.25 ± 0.10). Along with the kinetic lability causing rapid ligand exchange accompanied by NMR signal broadening and averaging and only small paramagnetic effects especially for the aminoacetate and to some extent for the propyl residue’s methylene signals is indicative of end-on binding by two carboxyl groups. Consequently, in [EuH_2_(DEGTA)]^+^ the ligand is not fully deprotonated and only two out of nine possible coordination sites are bound. The second species, forming at near-neutral pH at micromolar metal ion concentrations used for TRLFS, shows a much longer lifetime (924 ± 149 μs) and higher complex stability (log *K* = log β_110_ = 16.3 ± 0.1). At millimolar concentration as applied for NMR, this second complex exhibits strong paramagnetic effects mainly of pseudocontact interaction, qualitatively confirmed by quantum chemical calculation. This proves that all nine coordinating atoms (4 carboxyl and 3 ether oxygens and 2 amino nitrogens) are actually bound to the metal center. These calculations also have shown that the elongated backbone weakens particularly the Eu(III)–nitrogen bond which is the main reason why the fully deprotonated [Eu(DEGTA]^−^ complex only exists at sufficiently high pH values, i.e., as of commencing ammonium nitrogen deprotonation. Additionally, isostructural complexes were observed for Sm(III) and Cm(III) as inferred from NMR and TRLFS, respectively. A remarkably long luminescence lifetime (448 ± 12 μs) was also observed for [Cm(DEGTA]^−^ proving the 9-fold coordination for Cm(III) also. NMR spectra and DFT calculations (Figures S23 and S25 and Table S3) regarding La(III) complexation by DEGTA reveal much less stability in terms of both kinetics and thermodynamics as inferred from broad signals very much resembling free ligand and overall increased bond lengths for all coordinating atoms, respectively. The complex stability of the Cm complex (log *K* = log β_110_ = 17.9 ± 0.1) is slightly higher than that of the corresponding Eu complex, which is in line with previously published data for comparable systems (Table 3).^1^

Considering the reported behavior of related complexones NTA, EDTA, and EGTA, one could expect similar complexation behavior of DEGTA toward trivalent Eu(III) and Cm(III). However, its complexing properties are quite different. In agreement with the other three ligands, it forms the dominant 1:1 complex of the structure [ML]^−^ where *M* = Eu(III) or Cm(III) displaces the protons of all functional groups. Under comparable conditions, such as micromolar Eu(III) concentrations, this species occurs only at much higher pH values, viz. above pH 6.5 in contrast to pH 2–4 for the three other complexones. Furthermore, it forms a second 1:1 complex containing an only partly deprotonated ligand, which was not observed for NTA, EDTA, and EGTA, *ceteris paribus*. Lastly, the stability of the complex is weaker as one would maybe expect from the observed trend in the series as provoked by the steric hindrance of the extended backbone.

## 4 Material and Methods

### 4.1 Synthesis

1,13-Diamino-4,7,10-trioxatridecane (for synthesis, Sigma-Aldrich, 0.97 g, 1 equiv.) was dissolved and stirred in 20 mL of acetonitrile (99.8%, anhydrous, Sigma-Aldrich). To this solution, DIPEA (4.6 g, 8 equiv.) was added. Furthermore, ethyl bromoacetate (EBA, 98%, ABCR Chemicals, 3 g, 4 equiv.) was added dropwise, which resulted in an increase in the temperature of the solution. This reaction mixture was heated to reflux and stirred overnight. After 24 h, 2 more equivalents of EBA were added to the stirred solution. The progress of the reaction was monitored using mass spectrometry. After 3 days, the reaction was quenched using DI water. The ethyl-protected product was purified using normal phase column chromatography with *n*-hexane and ethyl acetate (gradient). To remove the ester groups, LiOH (>98%, Sigma-Aldrich, 0.5 g) in water was used. After deprotection, the product was collected using reverse phase column chromatography (water (0.1% TFA)/acetonitrile, gradient). The yield of the final product is 0.7 g (34%).

### 4.2 Starting Material and Stock Solutions

*Caution! Curium is a radioactive element with high radiotoxicity requiring special precautions for handling radioactive materials, and all studies were conducted in a laboratory dedicated to actinide research.*

Besides synthesized DEGTA, all chemicals were used as obtained. Stock solutions were prepared by weighing and dissolving appropriate amounts of EuCl_3_·6H_2_O (99.99%, Sigma-Aldrich, Taufkirchen, Germany) and H_4_EGTA (≥99%, Roth) in NaCl (99.5%, Roth) containing Milli-Q H_2_O (18.2 MW cm, Millipore, Merck, Darmstadt, Germany) and D_2_O (99.98% D, Deutero, Kastellaun, Germany) aqueous solutions. pH was adjusted with HCl (1.0, 0.1, and 0.01 M) and NaOH (1.0, 0.1, and 0.01 M) in D_2_O solutions, likewise, DCl and NaOD (both >99% D, Deutero), using a pH meter (inoLab pH 730, Xylem, Weilheim, Germany) equipped with a pH electrode (SCHOTT, BlueLine, SI Analytics, Mainz, Germany).

^248^Cm was obtained from the transplutonium element production facilities at Oak Ridge National Laboratory, Oak Ridge, TN, USA. Appropriate dilutions were made from a 295 μM Cm(ClO_4_)_3_ stock solution.

### 4.3 NMR Spectroscopy

NMR spectra were obtained at (25 ± 0.2) °C with Agilent DD2-600 and MR-400 systems, operating at 14.1 and 9.4 T, with corresponding ^1^H and ^13^C resonance frequencies of 599.8 and 150.8 MHz or 399.8 and 100.8 MHz, respectively, using 5 mm oneNMR probes. Ligand characterization also includes ^15^N NMR, recorded at 60.8 MHz (at 14.1 T). Chemical shifts are reported in parts per million relative to external TMS and liquid ammonia for ^1^H/^13^C and ^15^N, respectively.

Determination of ligand content in the white solid obtained from synthesis, purification, and lyophilization after neutralizing the highly viscous material yielded in an acidic medium, a stock solution was prepared. Therefore, a D_2_O solution containing an aliquot of the stock solution spiked with the internal standard TMSP-*d*_4_ (sodium 3-(trimethylsilyl)-2,2,3,3-tetradeuteropropionate) a ^1^H NMR spectrum was measured applying quantitative acquisition parameters, viz. excitation by a π/6 pulse (2.67 μs), a relaxation delay of 90 s, and an acquisition time of 5 s, upon accumulation of 16 individual spectra. Complemented by ICP-MS analyses, the material contains 11.78 and 1.16 mol of LiCl and NaCl per mole of DEGTA, respectively, as a remainder from synthesis and pH adjustments. In subsequent experiments, where required, these salt contents will accordingly be considered for background electrolyte ionic strength adjustments, especially in the case of determination of thermodynamic parameters.

For ligand characterization, D_2_O solutions of 30 mM in DEGTA were prepared at three pD values, viz. 1.0, 6.0, and 12.0. Corresponding ^1^H NMR spectra were measured by accumulating 8 scans, using 2 s of acquisition time and relaxation delay, respectively, applying a 2 s presaturation pulse on the water resonance for water signal suppression followed by a π/6 excitation pulse. For ^13^C{^1^H} NMR measurements, 128 scans were accumulated upon applying a 4 s relaxation delay after π/6 excitation pulse and 1 s acquisition time. Heteronuclear single-quantum coherence (HSQC) and heteronuclear multiple-bond correlation (HMBC) were accomplished using pulse sequences taking advantage of gradient-selection and adiabatic pulses. The latter as well as the NOESY spectra were obtained by applying a 1 s presaturation pulse for water signal suppression. ^1^H,^13^C-HSQC, ^1^H,^13^C-HMBC, and ^1^H,^15^N-HMBC spectra were acquired with 1024 × 256, 1024 × 512, and 1024 × 64 complex points in *F*_2_ and *F*_1_, 8, 16, and 64 transitions per *F*_1_ increment, and a relaxation delay of 1 s, respectively. For polarization transfer, (2*J*)^−1^ delays of 3.57 and 100.0 ms were opted, corresponding to 140 Hz ^1^*J*(H,C) in HSQC and 5 Hz ^n^*J*(H,C/N) in HMBC, respectively. The phase-sensitive NOESY spectrum of the pD 12 sample was acquired with a mixing time of 400 ms, 1024 × 256, complex points in *F*_2_ and *F*_1_, 8 transitions per F1 increment, and a relaxation delay of 1 s.

Preparation of samples as well as data acquisition and processing for p*K*_a_ determination was conducted according to the procedure and parameters as described elsewhere.^1^ Briefly, aqueous solutions containing 5% D_2_O per volume and the required amount of NaCl from a 1 M stock solution were added to finally obtain solutions containing a total background electrolyte (LiCl/NaCl) ionic strength of 100 mM and a DEGTA concentration of 1 mM. In total, 54 solutions were prepared in the range 0.57 ≤ pH ≤ 11.21 with increments of ca. 0.2 pH units.

For the characterization of DEGTA complexes of Eu(III) and Sm(III), D_2_O pD 8 solutions were prepared with metal ion and ligand concentrations of 20 and 30 mM, respectively. In the case of ^1^H and ^13^C{^1^H} NMR, 90° excitation pulses were used, applying 0.3 and 0.7 s as acquisition time and relaxation delay, accumulating 16 and 6000 scans, respectively. ^1^H as well as the 2D NMR spectra were measured applying 1 s presaturation pulse for water signal suppression. COSY (600 MHz), H,C-HSQC (400 MHz; 140 Hz ^1^*J*(H,C) corresponding to (2*J*)^−1^ = 3.57 ms), and NOESY (400 MHz, 50 ms mixing time) spectra were acquired in the case of Eu(III) with 2048 × 512, 2048 × 1024, and 2048 × 256 complex points in *F*_2_ and *F*_1_, 36, 80, and 128 transitions per *F*_1_ increment, and in the case of Sm(III) with 2048 × 256, 2048 × 128, and 2048 × 256 complex points in *F*_2_ and *F*_1_, 12, 72, and 64 transitions per *F*_1_ increment, using relaxation delays of 0.5 s, respectively.

Concentration- and pD-dependent Eu(III)–DEGTA solutions for NMR investigation were prepared by mixing appropriate aliquots of stock solutions of both DEGTA and EuCl_3_ in D_2_O, respectively, adjusting pD by using NaOD and DCl, according to the common pD = pH meter reading −0.4 in pure D_2_O. These solutions comprise five series, each of which applies 2 mM DEGTA. Two series varied the Eu(III) concentration (1.2, 10, 20, and 50 mM) at constant pD values of 5.1 and 6.1, respectively. Three series varied pD, two of which for excess Eu(III), viz. pD 3.1, 4.1, 5.1, and 6.1 at 50 mM [Eu] as well as pD 4.1, 5.1, and 6.1 at 10 mM [Eu], and one of which at ligand excess with pD 3.1, 4.1, 5.1, 6.1, 7.2, and 7.7 at 1.2 mM [Eu].

### 4.4 Luminescence Spectroscopy

Solutions were stirred in 10 mm path length Hellma Analytics 4 mL quartz cells. The cuvette was placed in a cuvette holder, which was connected via a light guide to a spectrograph (Andor, Belfast, UK, SR-303i-A). For recording the spectra, an ICCD (Andor iStar, DH320T-18U-63) was used. The excitation wavelength (Ekspla, Vilnius, Lithuania, NT230, ∼5 ns pulse) was 394 nm (grating: 300 mm^–1^). For Cm(III) luminescence spectroscopy, a pulsed flash lamp pumped Nd:YAG laser system (Powerlite Precision II 9020 laser equipped with a Green PANTHER EX OPO from Continuum, Santa Clara, CA, USA) was used. The laser system was equipped with a delay generator (Stanford Research Systems Inc., Sunnyvale, CA, USA, Model DG535). The luminescence spectra were detected using an optical multichannel analyzer system, consisting of an Andor Kymera 328i monochromator and spectrograph with gratings of 150, 300, 600, and 1200 lines per mm (Oxford Instruments, Abingdon, UK) and an Andor iStar ICCD camera (ICCD 05933, Andor). The excitation wavelength was 396 nm (grating: 300 mm^–1^).

### 4.5 Computational Details—DFT Calculations

For the geometry optimization, crystal structures served as starting geometries for the cases of [Eu(EDTA)(H_2_O)_3_]^−^ and [Eu(EGTA)(H_2_O)]^−^. Since for [Eu(DEGTA)]^−^ no crystal data could be obtained, a guess structure was modeled based on the ^1^H NMR spectrum incorporating the expected symmetry and ligating atoms. The optimizations were carried out on the density functional theory (DFT) level using TURBOMOLE 7.3.1^46^ applying the def-SVP basis set and the hybrid exchange correlation functional PBE0.^47^ The aqueous milieu was simulated via the conductor-like screening model (COSMO)^48^ with a dielectric constant of ε = 78.39. Scalar-relativistic effects present due to Eu were implicitly accounted for by the use of an effective core potential (ECP) for Eu, replacing the inner 28 electrons. After convergence, the structures were confirmed as energetic minima via numerical frequency analysis.

In the single-point DFT calculations (performed with ORCA 5.0.4),^49^ which were used for subsequent QTAIM analyses, all electrons are considered explicitly, and scalar relativistic effects are introduced through the Douglas–Kroll–Hess^50,51^ (DKH) formalism with appropriate basis sets (DKH-DEF2-TZVPP, Eu: SARC-DKH-TZVPP).^52^ The topological analysis itself was performed via AIMAll.^53^

The structure optimization for [Cm(DEGTA)]^−^ was carried out via ORCA (6.0.1) as well, since D4 London dispersion parameters^54^ (which are essential for a correct energetic description, especially for ligands that large) for elements beyond Am were not yet available in the TURBOMOLE version at hand. For the sake of convenience—regarding the implementation of ECPs in ORCA—and also for testing purposes, no ECP was used, but instead, an all-electron approach was applied (X2C-TZVPALL^55^ + SARC-DKH-TZVPP (Cm)/PBE0/CPCM(h2o).^56^ A comparison of the [Eu(DEGTA)]^−^ structure calculated via both TURBOMOLE and ORCA, using the procedures described, showed very good agreement (see Figure S25), suggesting a reasonable comparability for the [Cm(DEGTA)]^−^ structure.

### 4.6 Computational Details—Conformer Search and Energetic Sorting

The conformer ensemble of [Eu(DEGTA)]^−^ was generated using CREST with the GFN-FF,^57^ yielding 590 initial unique conformers. Subsequent energetic sorting was performed via CENSO^58^ (interfacing with ORCA) at the r^2^SCAN-3c^59^/def2-TZVP level, resulting in a total of four conformers below a threshold of 3.5 kcal/mol with similar structural motifs. The lowest energy was optimized once again at the def-SVP/PBE0/COSMO[h2o] level.

### 4.7 Data Processing Software

For calculating complex formation constants extrapolated to zero ionic strength by means of SIT, the program Aqua Solution Software (Acadsoft, York, UK)^60^ was used. Speciation calculations of different ligands were carried out with PHREEQC Interactive, version 3.7.3-15968. NMR spectra were processed with MestReNova, version 6.0.2., Mestrelab Research S.L., Santiago de Compostela, Spain.^61^ The PARAFAC-assisted evaluation of luminescence spectra is described elsewhere.^22,62^ Creation of graphs for numerical data visualization and data fitting by a nonlinear sigmoidal dose–response fit algorithm was performed with Origin 2019, version 9.6.0.172, OriginLab Corporation, Northampton, MA, USA.

## Supporting Information Available

Ligand characterization by ^1^H, ^13^C, and ^15^N NMR spectroscopy, p*K*_a_ determination by means of ^1^H NMR-detected pH-titration, PARAFAC results of the complexation of DEGTA with Eu(III) in dependence of ligand concentration; structure determination of the complexation of DEGTA with Ln(III) by NMR spectroscopy, QTAIM analysis, Δ*G* comparison of low-lying conformers of [Eu(DEGTA)]^−^; complex formation constants for Eu(III) complexation by DEGTA, NMR chemical shift values of [Eu(DEGTA)]^−^ and [Sm(DEGTA)]^−^; distances and delocalization indices between atoms from the DEGTA 1:1 complexes with La(III) and Eu(III), simulated IR (PDF)
