# Supplementary material for: Synthesis of Nonadentate Ligand Diethylene Glycol-Bis(3-Aminopropyl Ether)-N,N,N′,N′-Tetraacetic Acid DEGTA and Its Complexation Behavior toward Trivalent Lanthanides and Actinides
Source: Inorg Chem. 2025 Feb 28;64(10):5014–28. doi: 10.1021/acs.inorgchem.4c05049 (PMC11920954; doi:10.1021/acs.inorgchem.4c05049)
Supplement: Supplementary file 1 — ic4c05049_si_001.pdf [file ic4c05049_si_001.pdf]

Supporting Information

Synthesis of nonadentate ligand diethylene glycol-bis(3-aminopropyl ether)-*N*,*N*,*N′*,*N′*-tetraacetic acid DEGTA and its complexation behavior towards trivalent lanthanides and actinides

Sebastian Friedrich[1], Adrian Näder[1], Björn Drobot[1], Jerome Kretzschmar[1], Thorsten Stumpf[1], Astrid Barkleit[1*]

[1]Helmholtz-Zentrum Dresden–Rossendorf, Institute of Resource Ecology, 01328 Dresden, Germany.
*Corresponding author. Email: a.barkleit@hzdr.de

Ligand characterization by ^{1}H, ^{13}C, and ^{15}N NMR spectroscopy

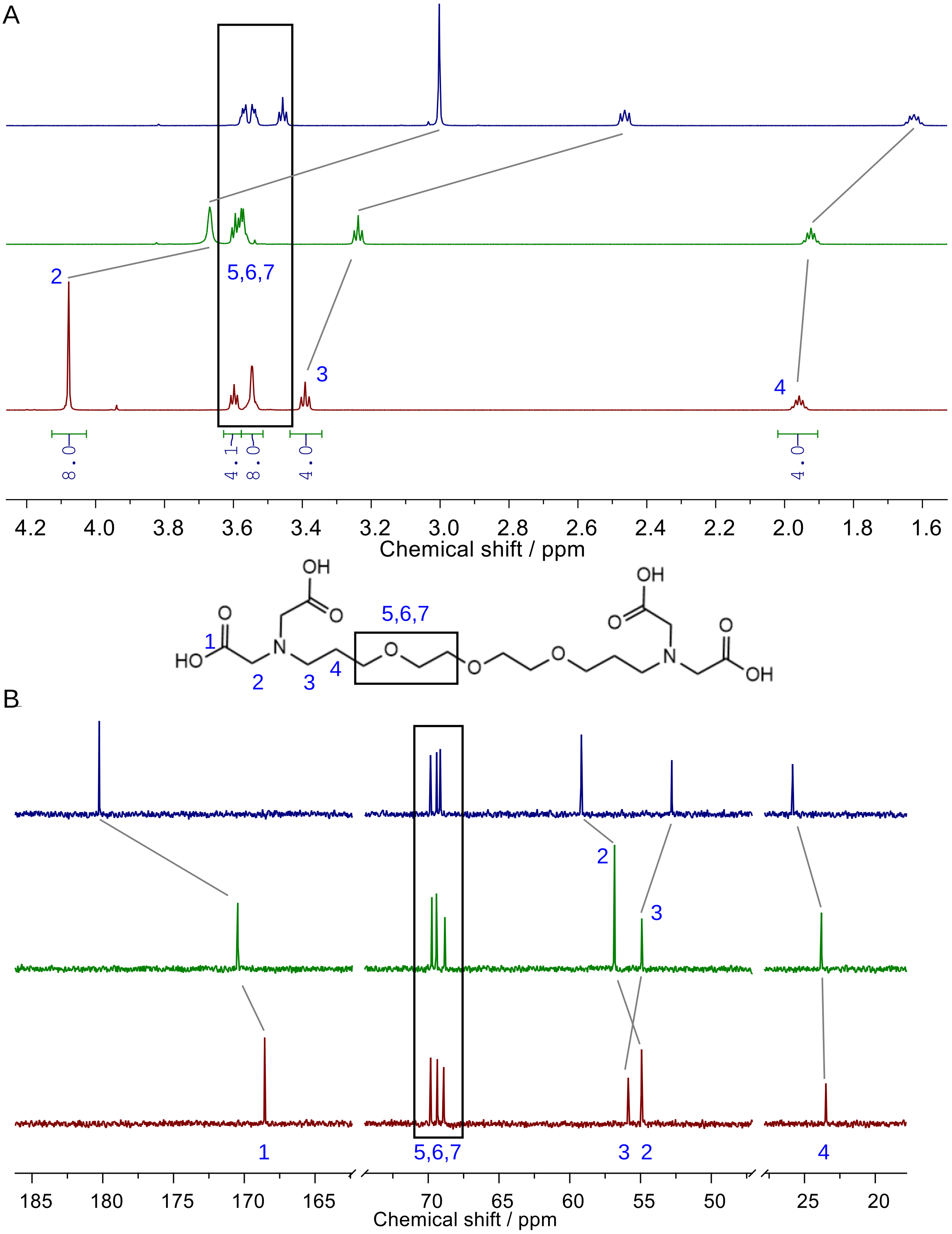

Figure S1: ^{1}H (A) and ^{13}C NMR (B) spectra obtained from 30 mM solutions of DEGTA in D_2O at pD values of 1.0 (bottom spectra, red), 6.0 (middle spectra, green), and 12.0 (top spectra, blue). Indicated regions comprise signals of ambiguous assignment owing to overlap and changing position upon varying pH. Unambiguous, pD-dependent signal assignment is achieved by the 2D-NMR (*vide infra*).

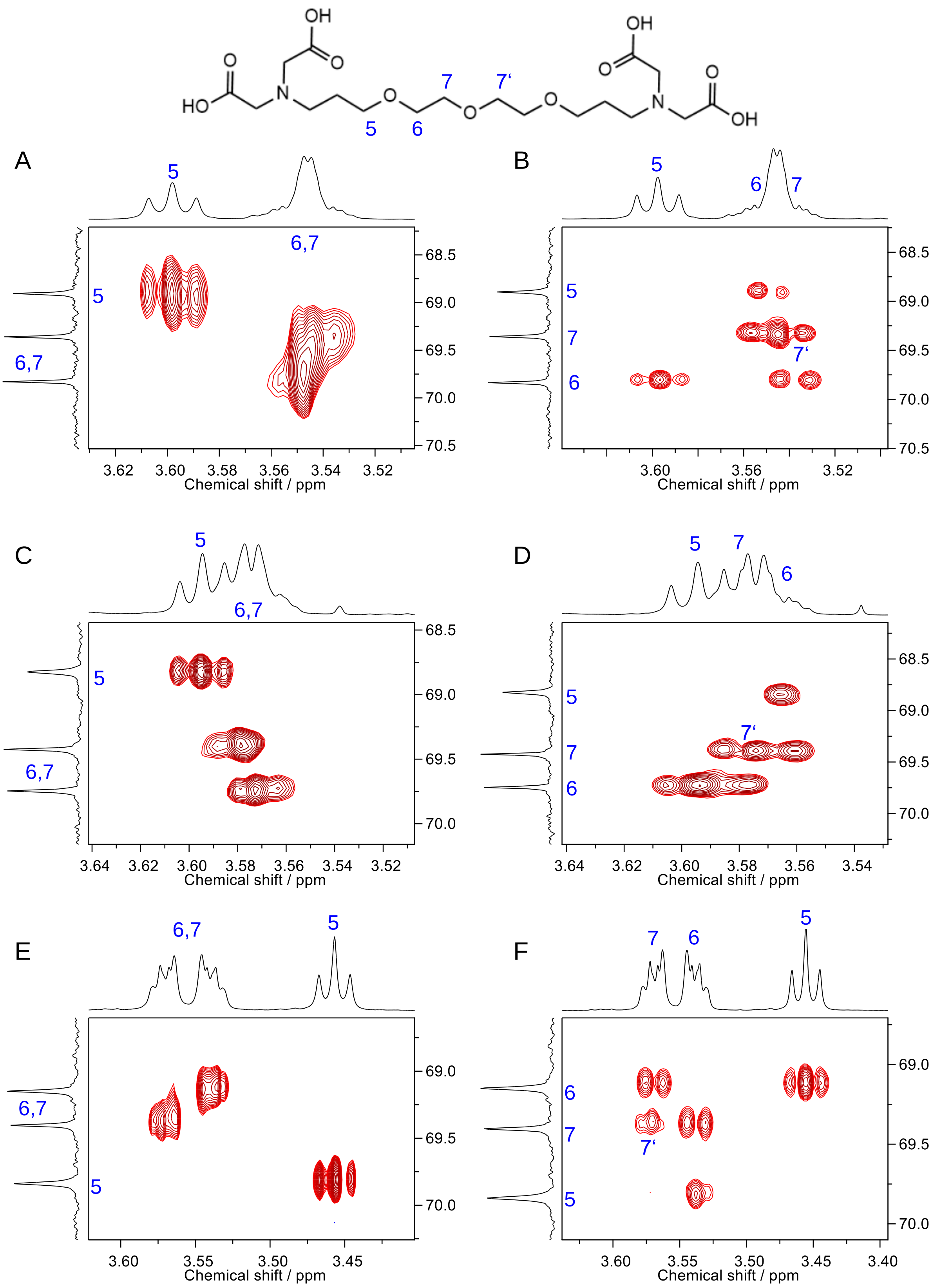

Figure S2: ^{1}H,^{13}C-HSQC NMR spectra (left panel) and ^{1}H,^{13}C-HMBC NMR spectra (right panel) obtained from 30 mM solutions of DEGTA in D_2O at pD values of 1.0 (top row), 6.0 (middle row), and 12.0 (bottom row), showing the regions of signals associated with the CH_2 groups adjacent to oxygen atoms, i.e., those in sites 5, 6, and 7.

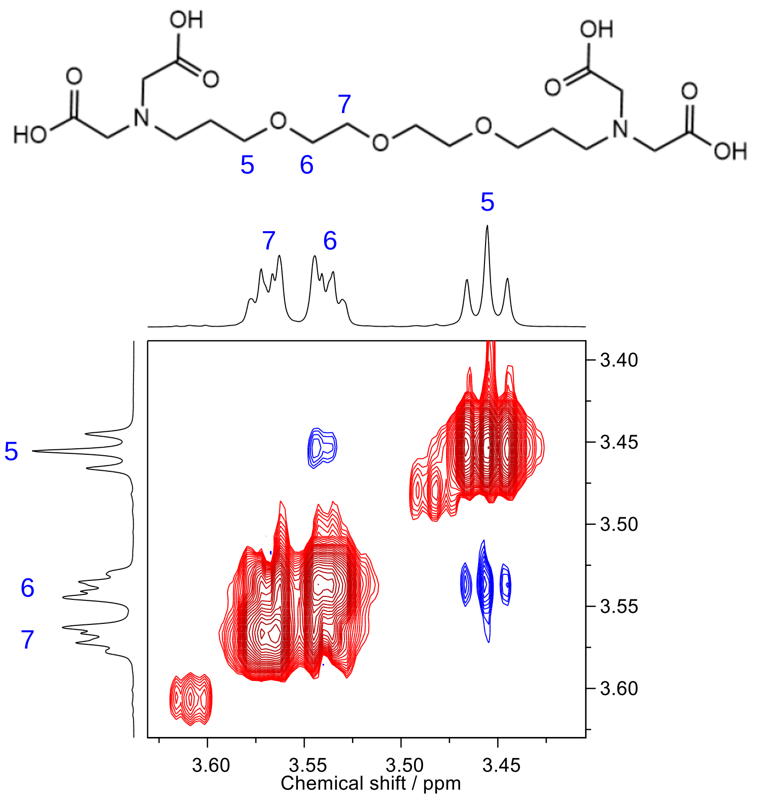

Figure S3: $^{1}H,^{1}H$-NOESY NMR spectrum of a 30 mM solution of DEGTA in D_2O at pD 12.0, showing the region of signals associated with the CH_2 groups adjacent to oxygen atoms, i.e., those in sites 5, 6, and 7.

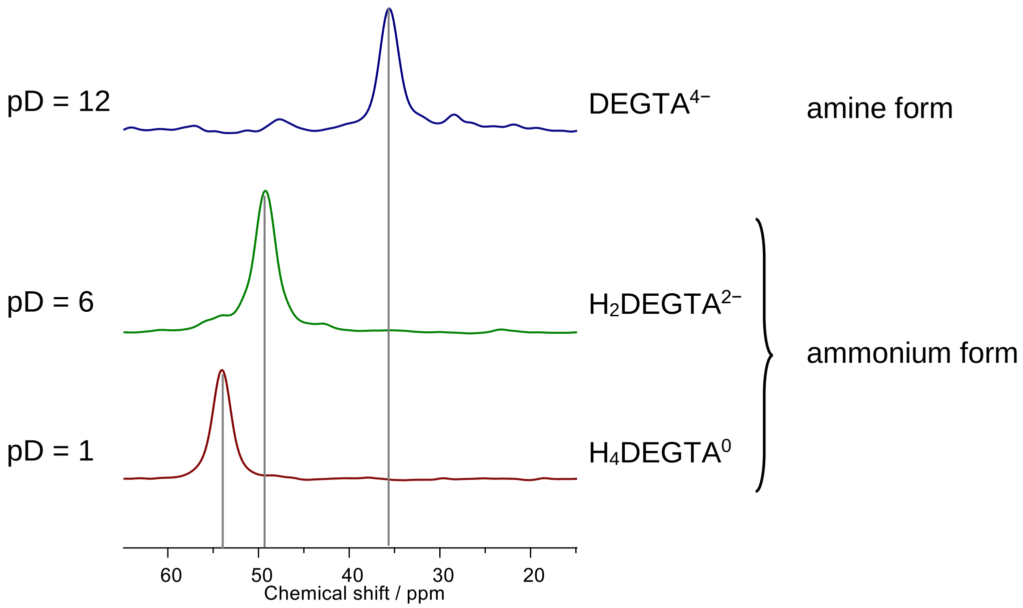

Figure S4: ^{15}N NMR signals obtained as F_1 projections from $^{1}H,^{15}N$-HMBC spectra of 30 mM solutions of DEGTA in D_2O at pD values stated with the spectra.

pK_a determination by means of ^{1}H NMR-detected pH-titration

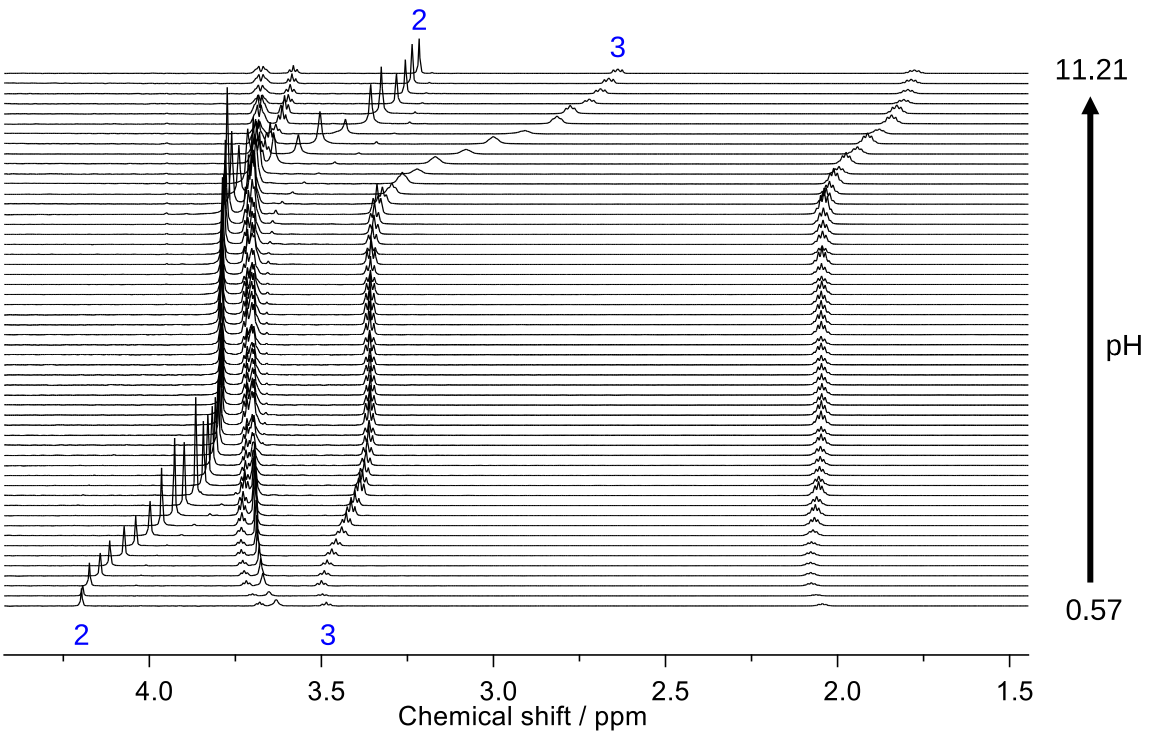

Figure S5: ^{1}H NMR spectra obtained from a pH-titration series in the range 0.57 ≤ pH ≤ 11.21 incremented by <0.25 pH units. The aqueous solution contained 10% per volume D_2O and a constant background electrolyte concentration of 0.1 M, and 1.0 mM DEGTA.

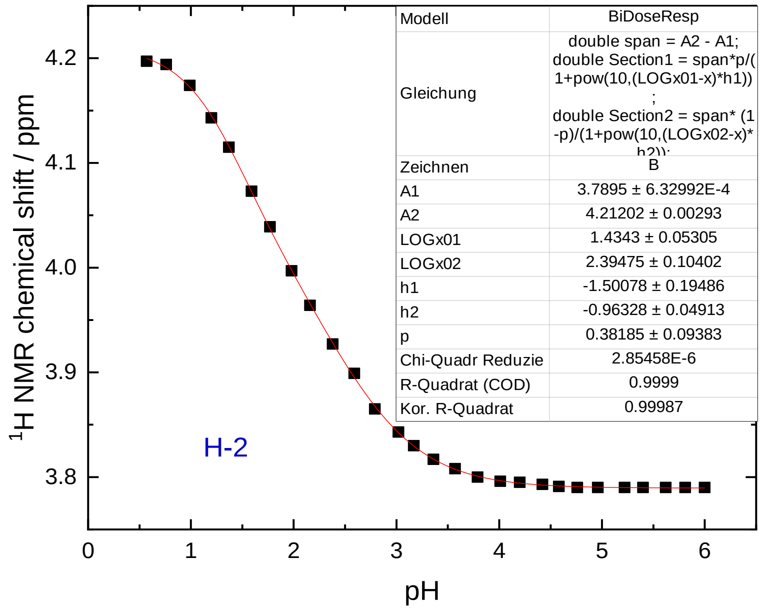

Figure S6: Plot showing the pH-dependent ^{1}H chemical shift values of the CH_2 groups adjacent to the carboxylic groups (data points) along with a sigmoidal bi-dose–response fit (red line) for corresponding pK_a determination.

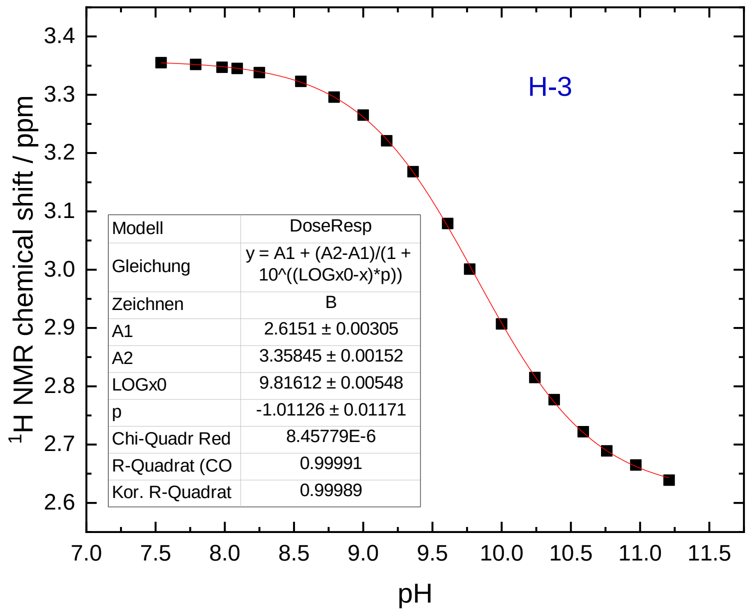

Figure S7: Plot showing the pH-dependent ^{1}H chemical shift values of the alkyl chain's amino nitrogen-neighboring CH_2 (data points) along with a sigmoidal dose–response fit (red line) for corresponding pK_a determination.

Complexation of DEGTA with Ln(III) and Cm(III)

Time-resolved laser-induced fluorescence spectroscopy (TRLFS)

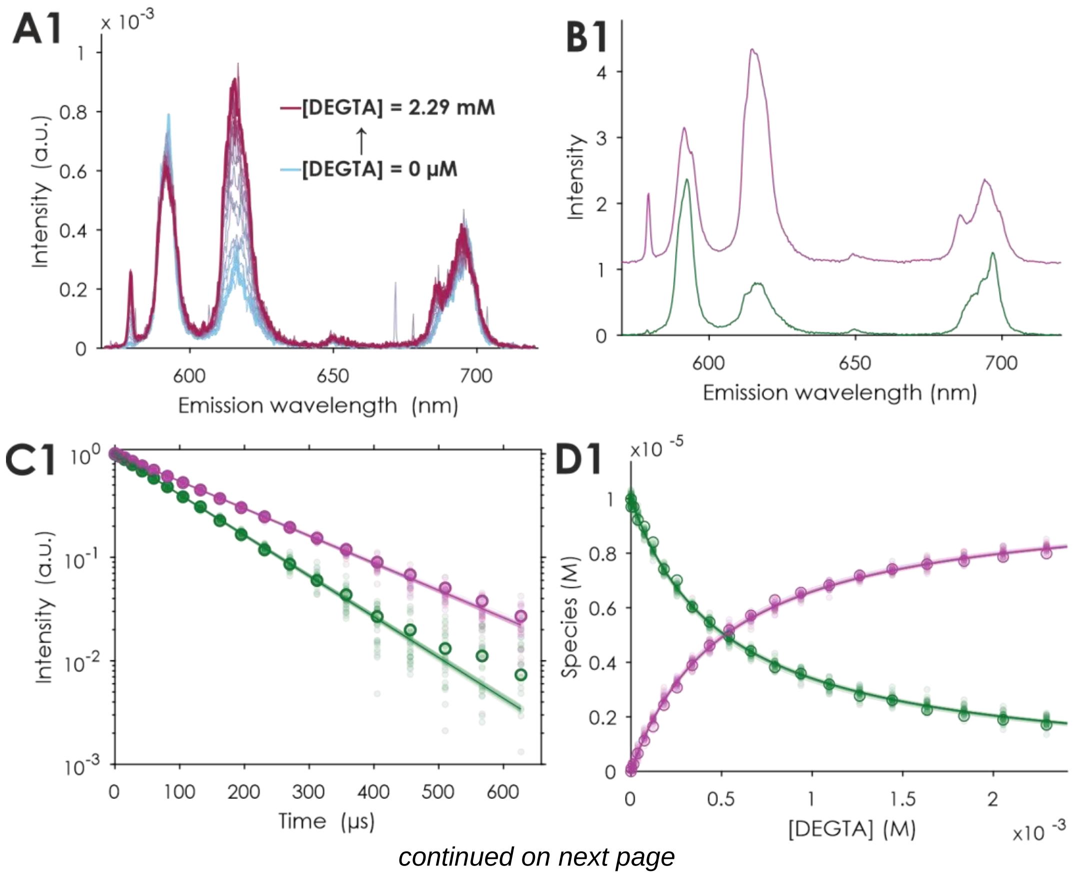

continued on next page

…continued

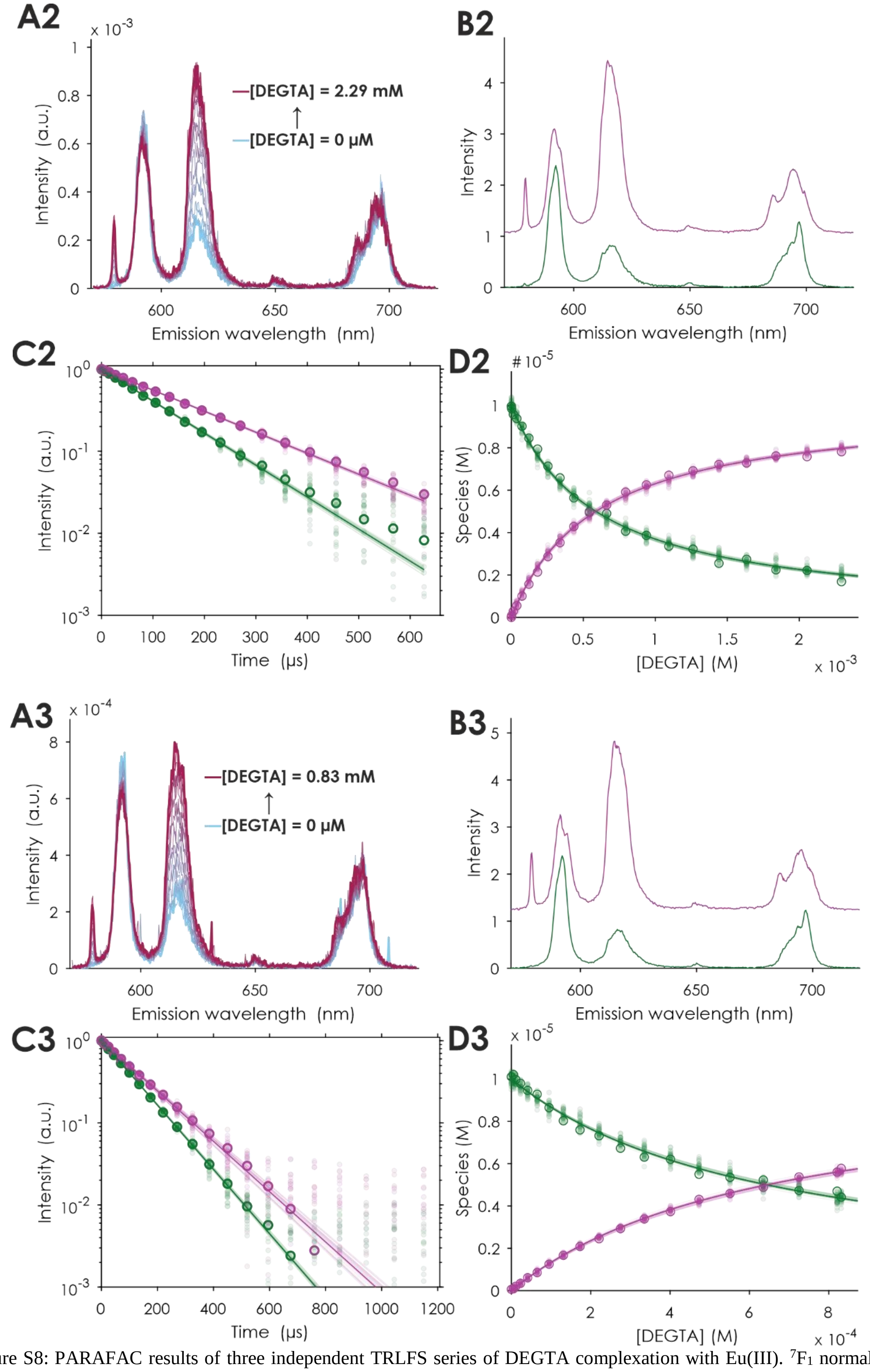

Figure S8: PARAFAC results of three independent TRLFS series of DEGTA complexation with Eu(III). $^7F_1$ normalized emission spectra at t = 0 μs (A), extracted single-component emission spectra (B), luminescence decays (C), and quantum yield-corrected PARAFAC distributions (symbols) and corresponding speciation (lines) (D). [Eu(III)] = 10 μM, [DEGTA] = 0 – 2.29 mM (**A** and **B**) or 0 – 0.83 mM (**C**), *I* (Li/NaCl) = 0.1 M, pH = 4.0 ± 0.1. The shaded data points were artificially created to be used in a Monte Carlo approach for the error estimation of the underlying model.

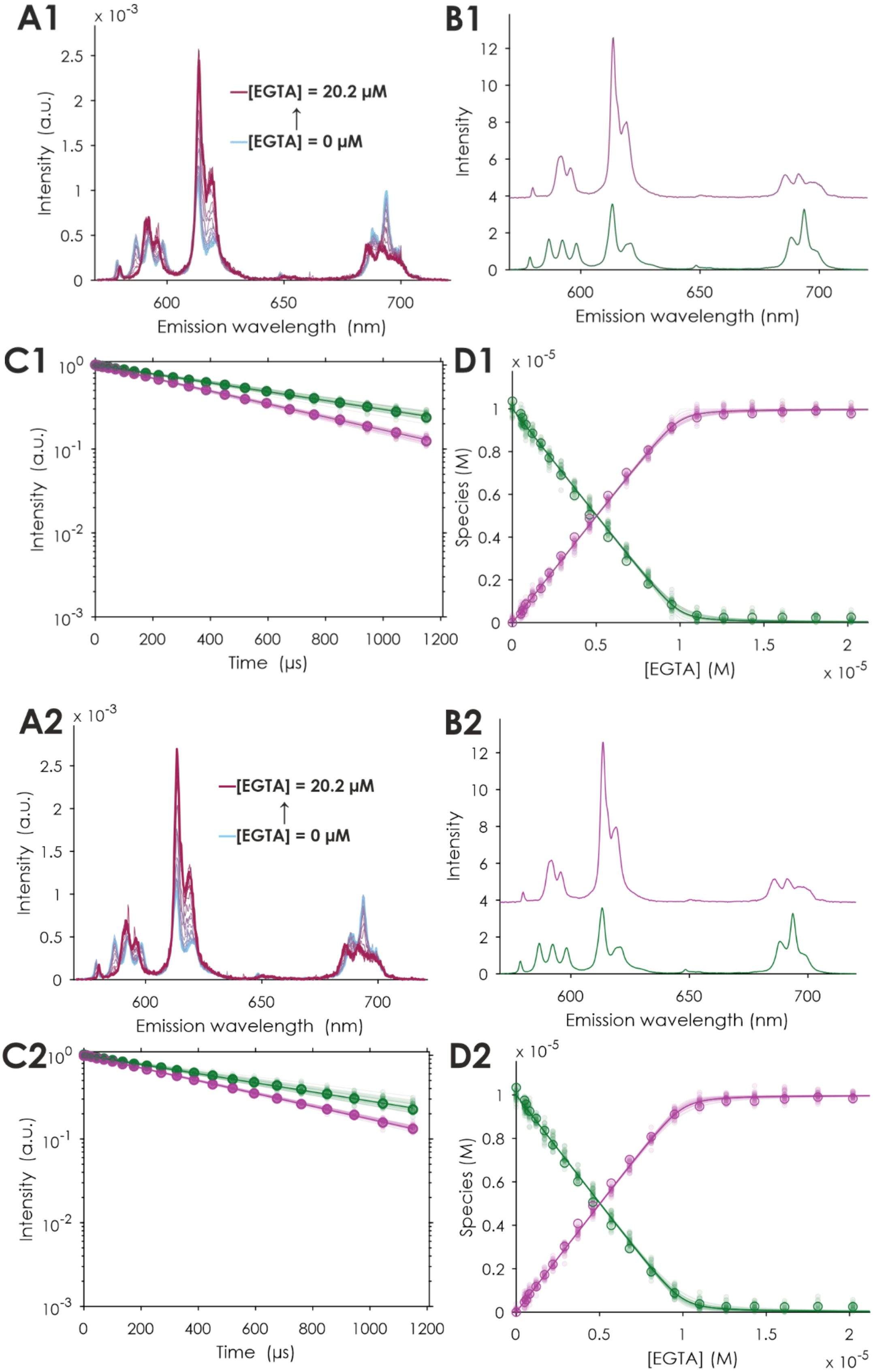

continued on next page…

…continued

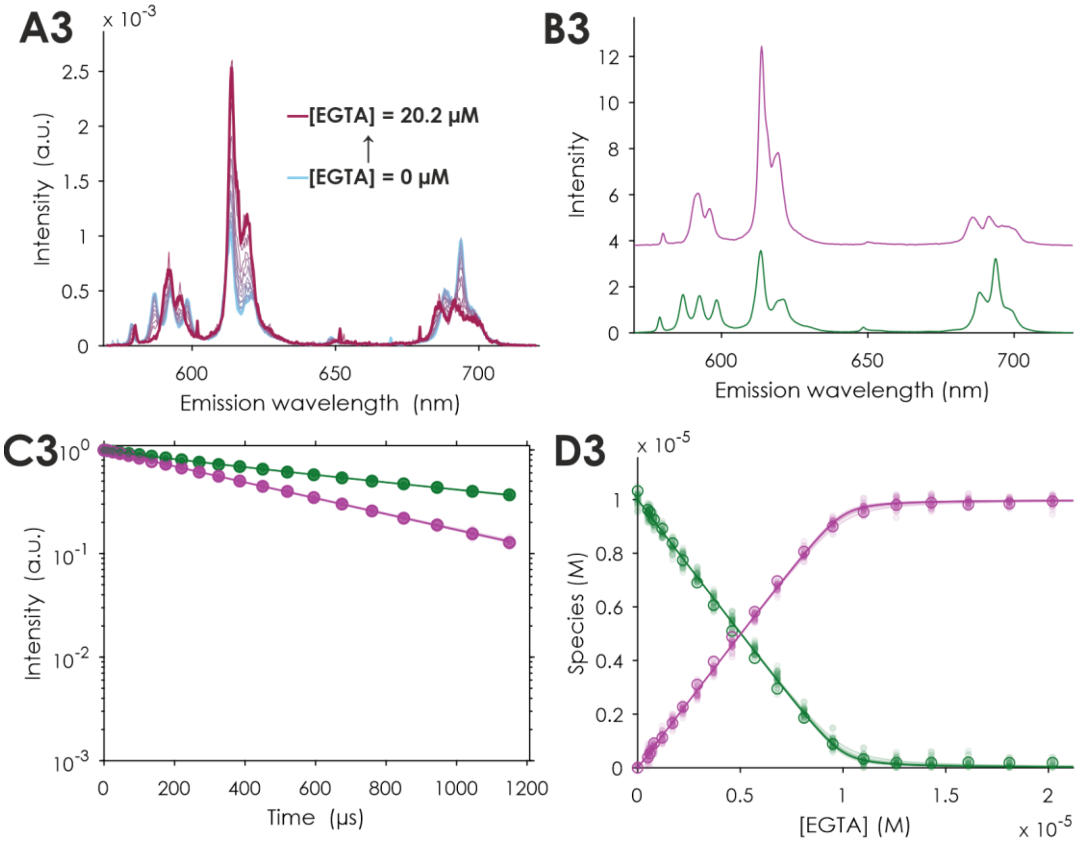

Figure S9: PARAFAC results of three independent TRLFS series of EGTA competition against DEGTA complexing Eu(III). $^7F_1$ normalized emission spectra at t = 0 µs (A), extracted single-component emission spectra (B), luminescence decays (C), and quantum yield-corrected PARAFAC distributions (symbols) and corresponding speciation (lines) (D). [Eu(III)] = 10 µM, [DEGTA] = 20 µM, [EGTA] = 0 – 20.2 µM, [TRIS] = 1 mM, I (Li/NaCl) = 0.1 M, pH = 7.5 ± 0.1. The shaded data points were artificially created to be used in a Monte Carlo approach for the error estimation of the underlying model.

Table S1: Logarithmic complex formation constant individual data of TRLFS measurements of Eu(III) complexes with DEGTA, obtained from triplicate experiments.

Species	Run		
	1	2	3
$[EuH_2(DEGTA)]^+$ (log K)	3.2	3.3	3.25
$[Eu(DEGTA)]^-$ (log β)	16.2	16.3	16.3

Structure determination

Table S2: ^{1}H and ^{13}C NMR chemical shift values (in ppm) of $[Eu(DEGTA)]^-$ and $[Sm(DEGTA)]^-$.

	pH 1	pH 6	pH 12	$[Eu(DEGTA)]^-$ (pD 8)	$[Sm(DEGTA)]^-$ (pD 8)
C1	168.6	170.5	180.2	1 188.8<br>1' 151.6	1 190.6<br>1' 187.3
C2	54.9	56.8	59.2	2 76.8<br>2' 57.8	2 71.7<br>2' 70.0
C3	55.9	54.9	52.8	94.1	53.9
C4	23.5	23.8	25.8	55.3	18.7
C5	68.9	68.8	69.9	141.9	65.34
C6	69.8	69.7	69.1	107.9	66.1
C7	69.4	69.4	69.4	77.5	71.8
H2	4.08	3.67	3.00	2a −0.8, 2b −14.5<br>2'a 2.3, 2'b −19.4	2a 5.12, 2b5.04<br>2'a 1.98, 2'b 1.94
H3	3.39	3.24	2.46	3a 26.5, 3b 1.2	3a 0.63, 3b −1.35
H4	1.96	1.92	1.62	4a 23.8, 4b 20.8	4a −1.59, 4b −1.92
H5	3.60	3.59	3.46	5a 53.2, 5b 26.1	5a −1.09, 5b −4.78
H6	3.55	3.57	3.54	6a 14.0, 6b 11.9	6a 5.20, 6b 3.94
H7	3.54	3.58	3.57	7a −6.2, 7b −6.6	7a 4.74, 7b 4.56

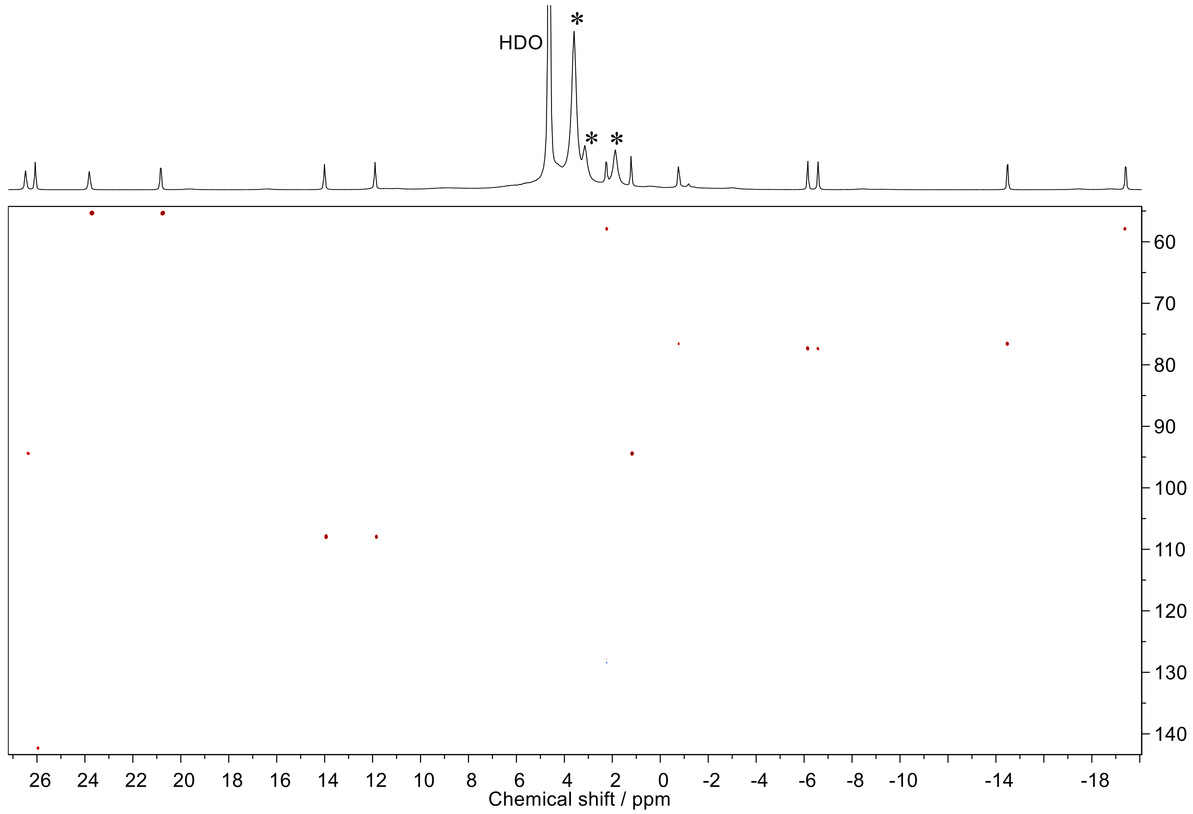

Figure S10: ^{1}H,^{13}C-HSQC spectrum of the Eu(III)–DEGTA complex in D_2O solution containing 20 mM Eu(III) and excess ligand at pD 8. Asterisks indicate signals due to excess ligand.

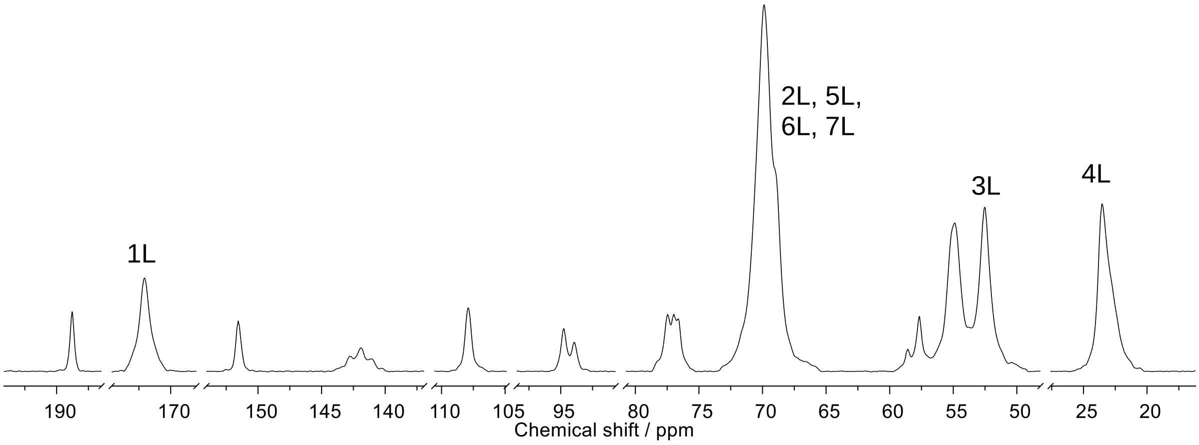

Figure S11: $^{13}C\{^{1}H\}$ spectrum (lb = 50 Hz) of the Eu(III)–DEGTA complex in D_2O solution containing 20 mM Eu(III) and excess ligand at pD 8. Indicated signals refer to carbon sites in the free ligand (L) and complement signal assignment shown in Figure 8C.

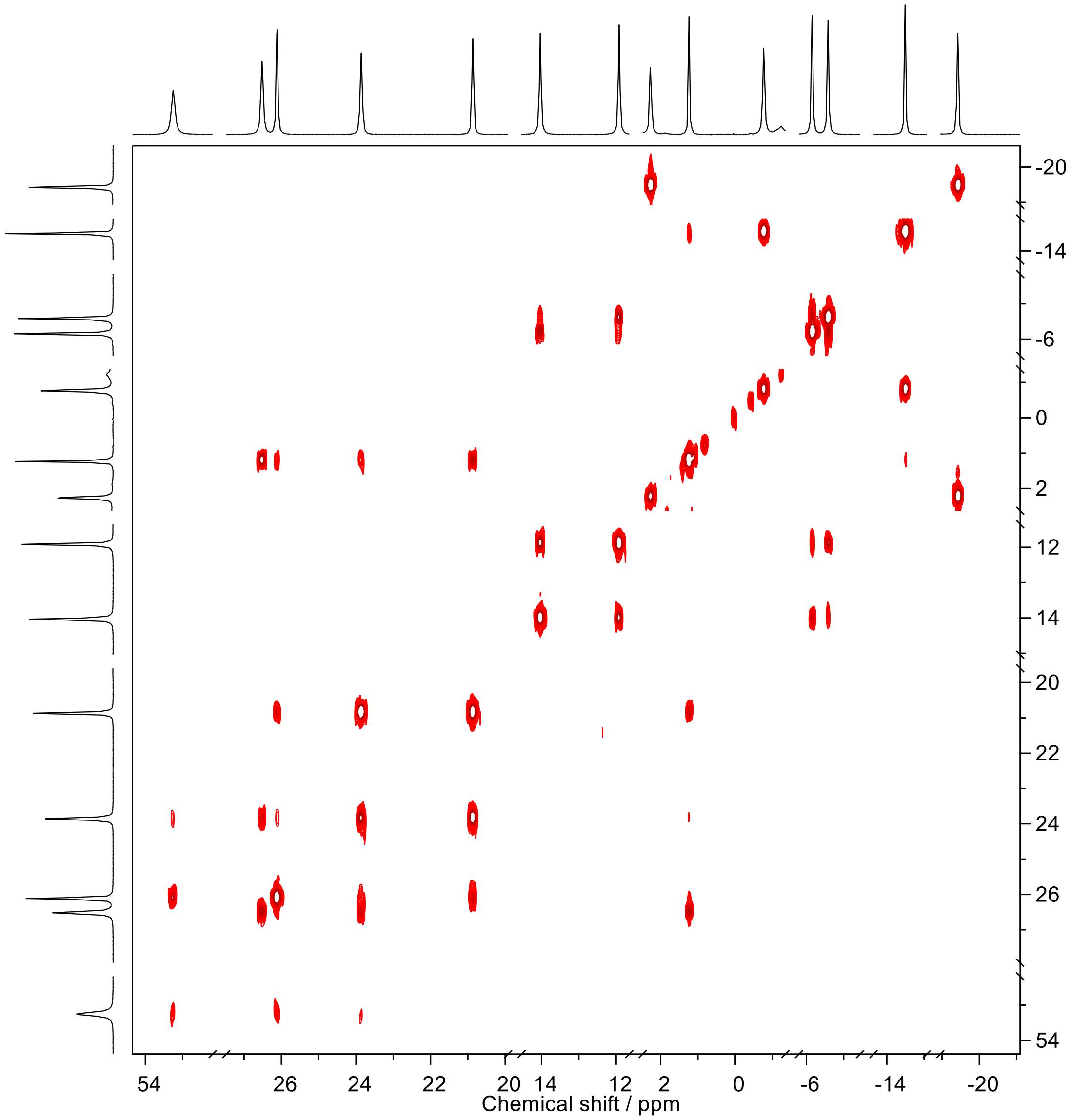

Figure S12: COSY spectrum of the Eu(III)–DEGTA complex in D_2O solution containing 20 mM Eu(III) and excess ligand at pD 8. For clarity, only complex-associated spectral regions are shown.

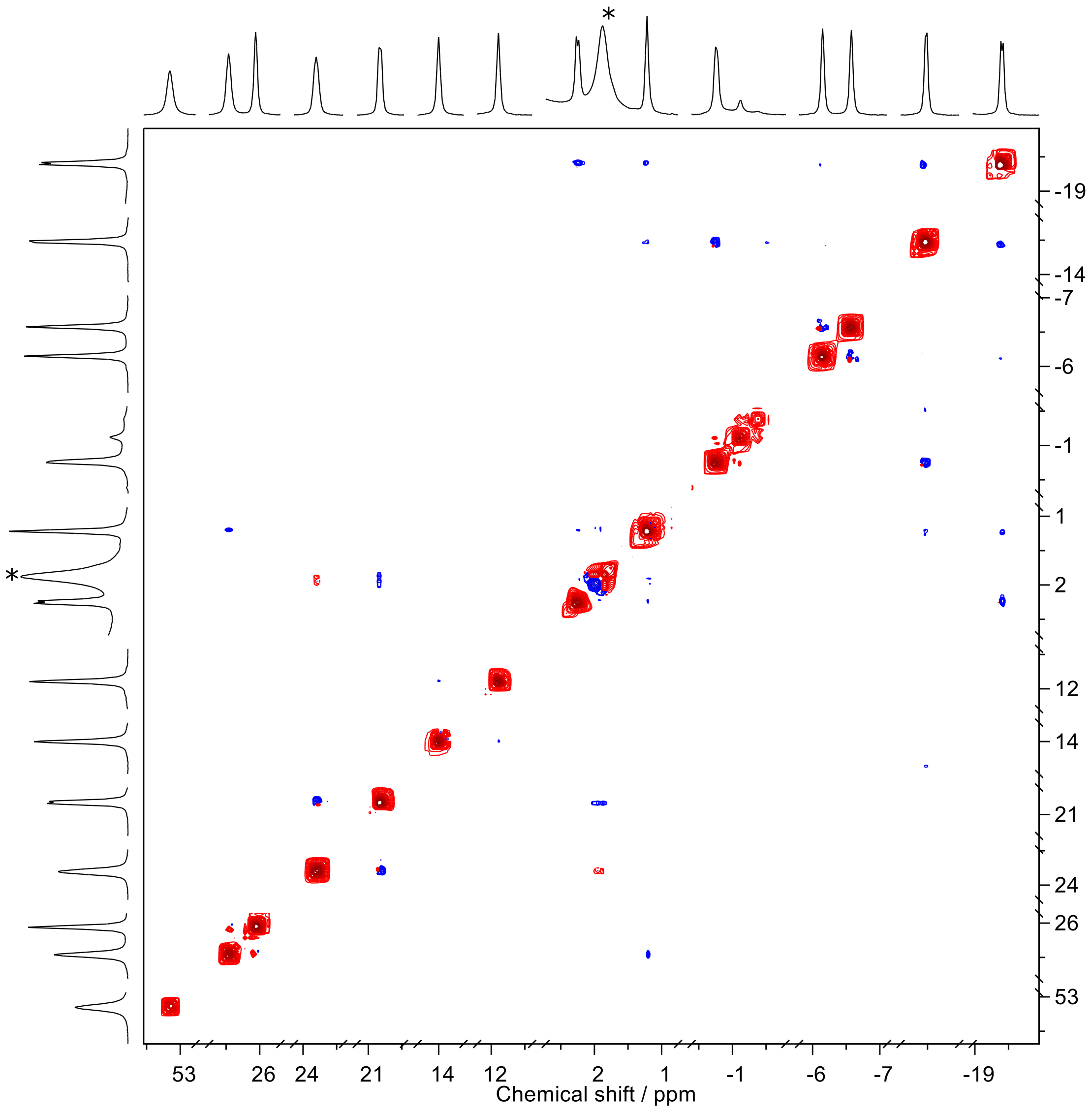

Figure S13: NOESY spectrum (mixing time 50 ms) of the Eu(III)–DEGTA complex in D_2O solution containing 20 mM Eu(III) and excess ligand at pD 8. For clarity, only complex-associated spectral regions are shown. The asterisk indicates a signal due to excess ligand.

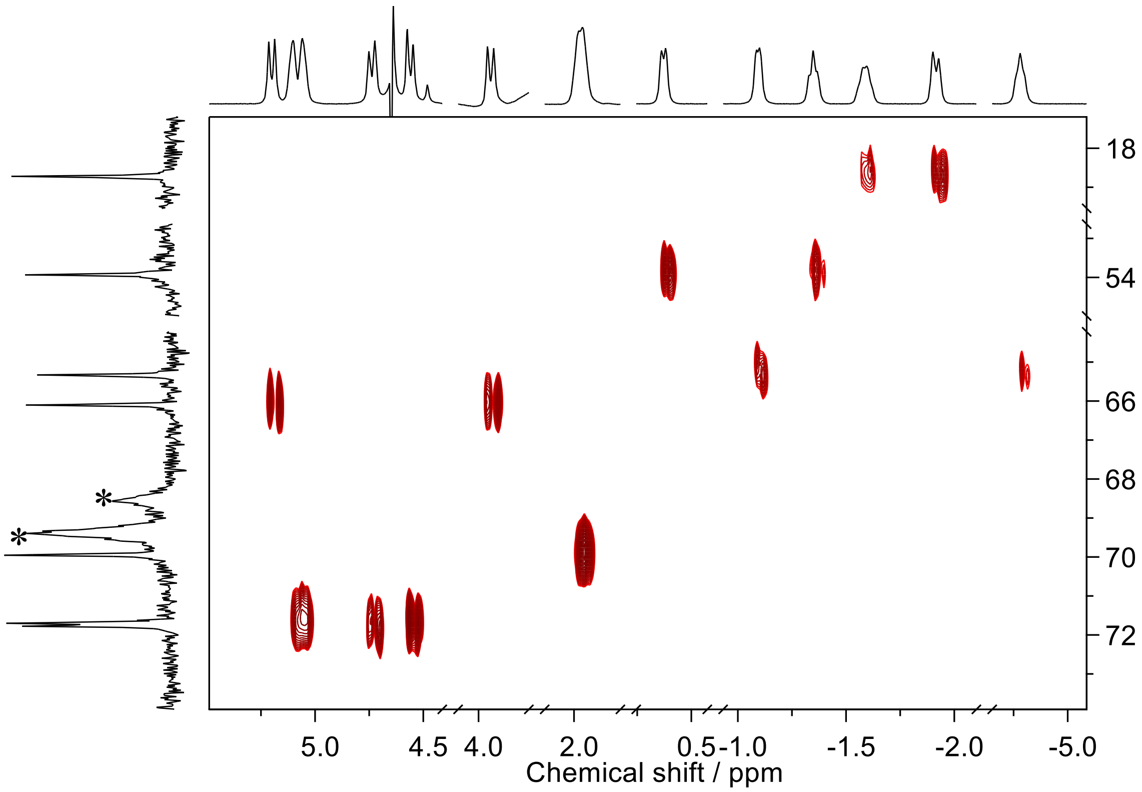

Figure S14: $^{1}H,^{13}C$-HSQC spectrum of the Sm(III)–DEGTA complex in D_2O solution containing 20 mM Sm(III) and excess ligand at pD 8. Asterisks indicate signals due to excess ligand.

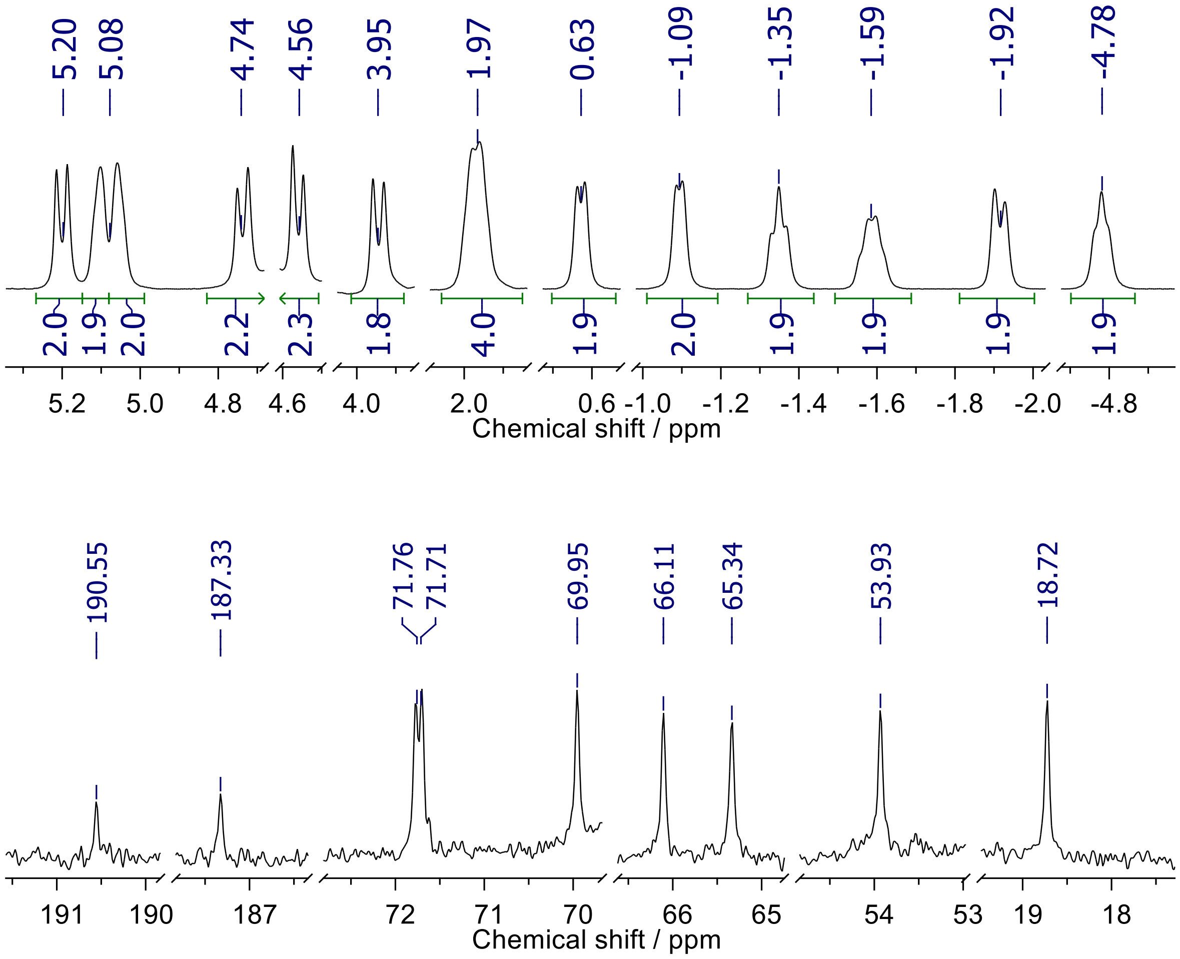

Figure S15: ^{1}H NMR (top) and ^{13}C{^{1}H} NMR spectrum (bottom) of the Sm(III)–DEGTA complex in D_2O solution containing 20 mM Sm(III) and excess ligand at pD 8. For clarity, only complex-associated spectral regions are shown.

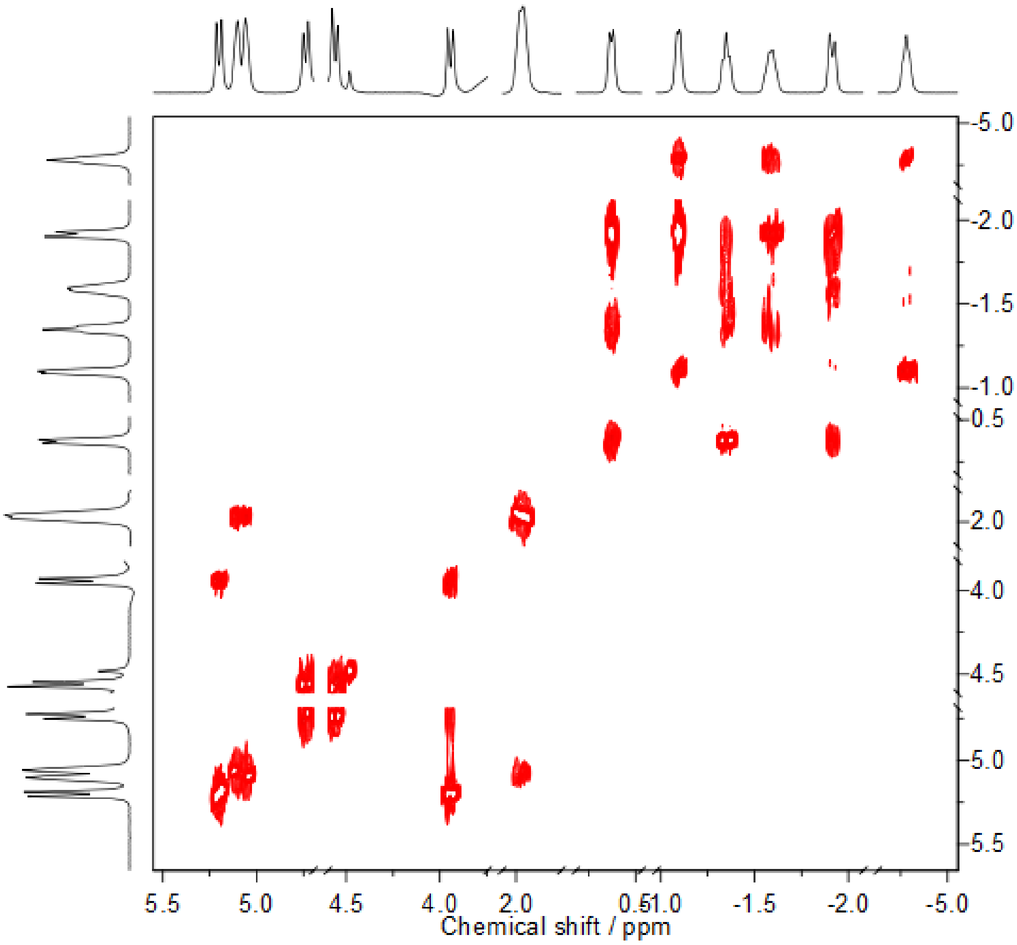

Figure S16: COSY spectrum of the Sm(III)–DEGTA complex in D_2O solution containing 20 mM Sm(III) and excess ligand at pD 8. For clarity, only complex-associated spectral regions are shown.

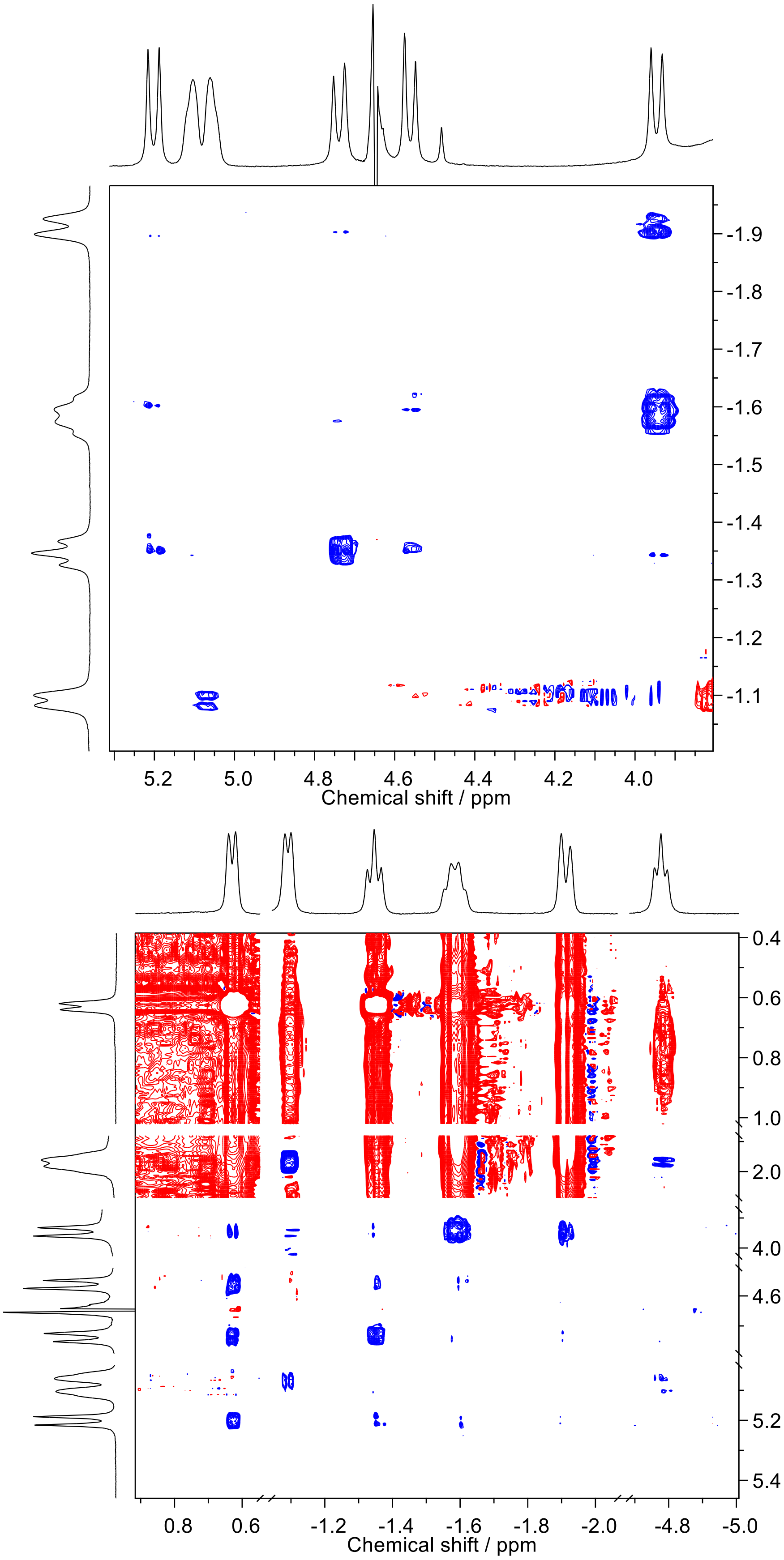

Figure S17: Two selected expansions of the NOESY spectrum (mixing time 50 ms) of the Sm(III)–DEGTA complex in D_2O solution containing 20 mM Sm(III) and excess ligand at pD 8. For clarity, only complex-associated spectral regions are shown.

Concentration- and pD-dependent NMR-titration series of DEGTA with Eu(III)

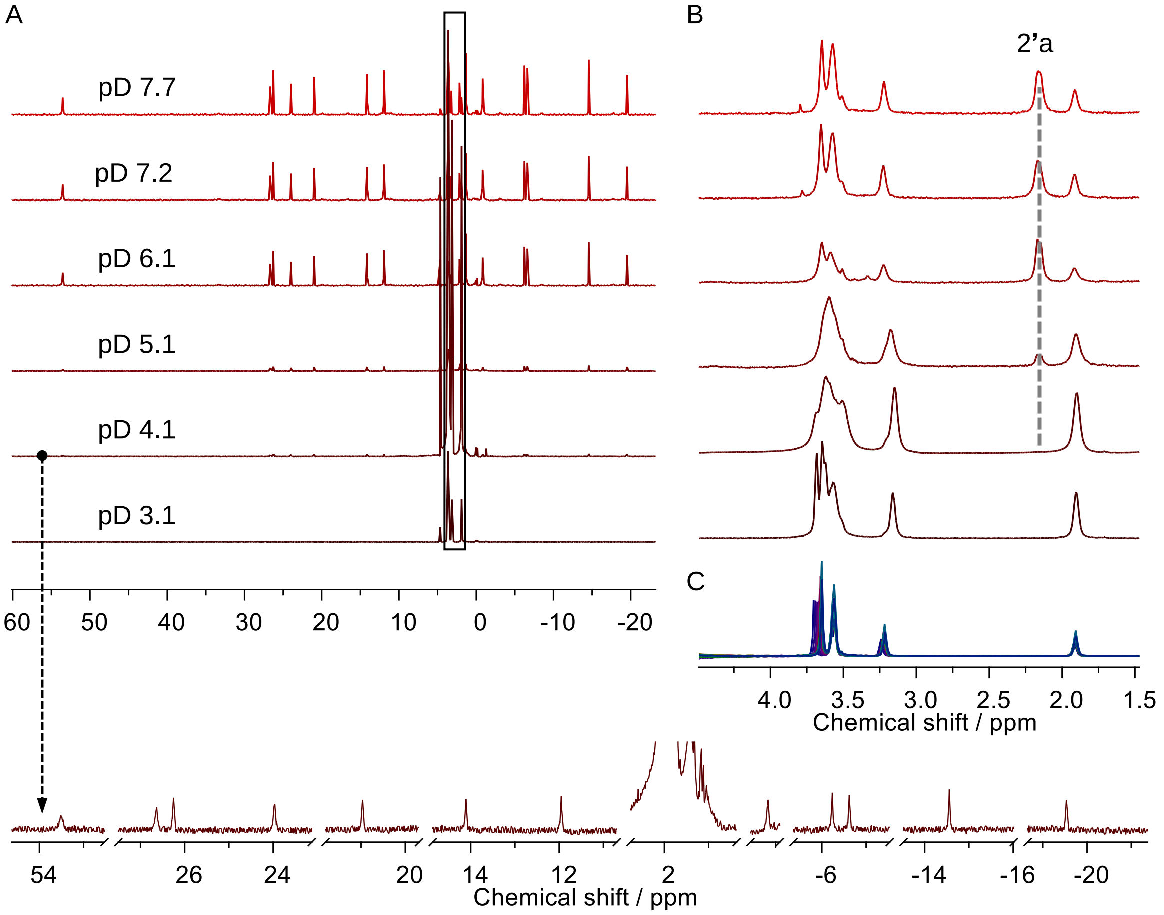

Figure S18: (A) ^{1}H NMR spectra of D_2O solutions 1.2 mM in Eu(III), 2 mM in DEGTA, obtained at pD values stated with the spectra. (B) Magnifications of a selected spectral region from (A). (C) Superposition of ^{1}H NMR spectra of the ligand pH-titration series comprising 27 spectra in the range 3.0 < pH < 7.8.

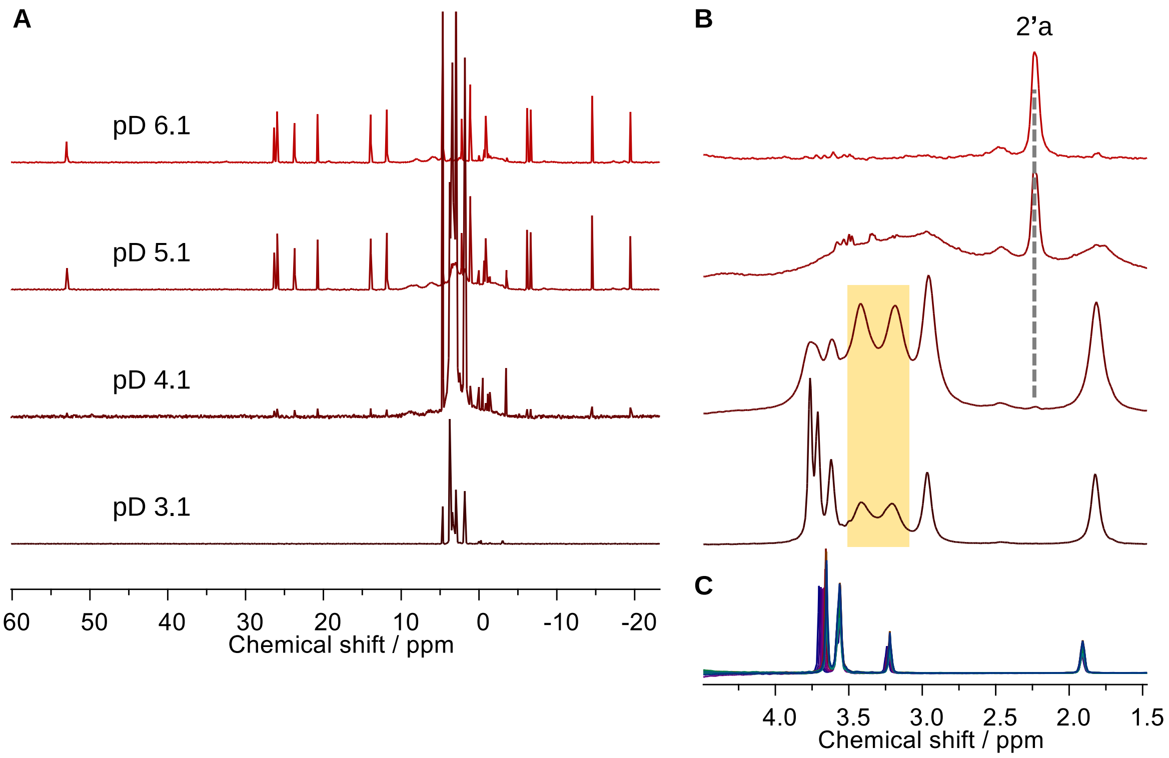

Figure S19: (A) ^{1}H NMR spectra of D_2O solutions 50 mM in Eu(III), 2 mM in DEGTA, obtained at pD values stated with the spectra. (B) Magnifications of a selected spectral region from (A). (C) Superposition of ^{1}H NMR blank spectra of the ligand pH-titration series comprising 17 spectra in the range $3.0 < pH < 6.2$.

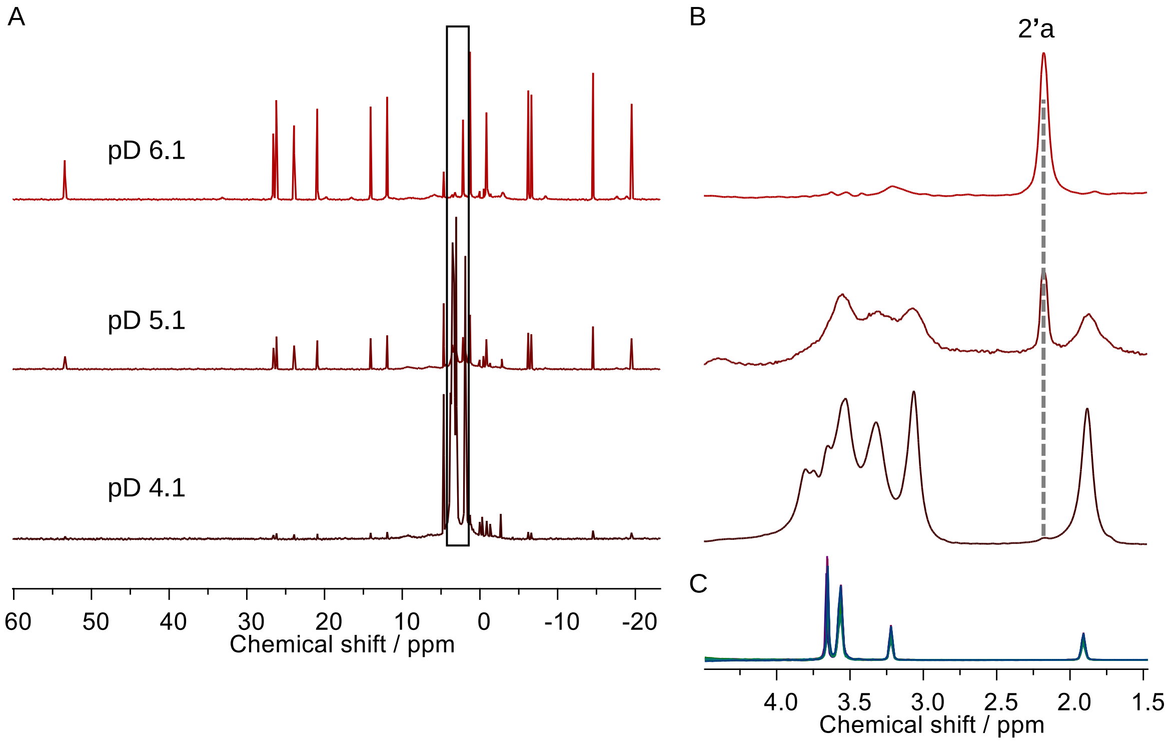

Figure S20: (A) ^{1}H NMR spectra of D_2O solutions 10 mM in Eu(III), 2 mM in DEGTA, obtained at pD values stated with the spectra. (B) Magnifications of a selected spectral region from (A). (C) Superposition of ^{1}H NMR blank spectra of the ligand pH-titration series comprising 13 spectra in the range $3.8 < pH < 6.2$

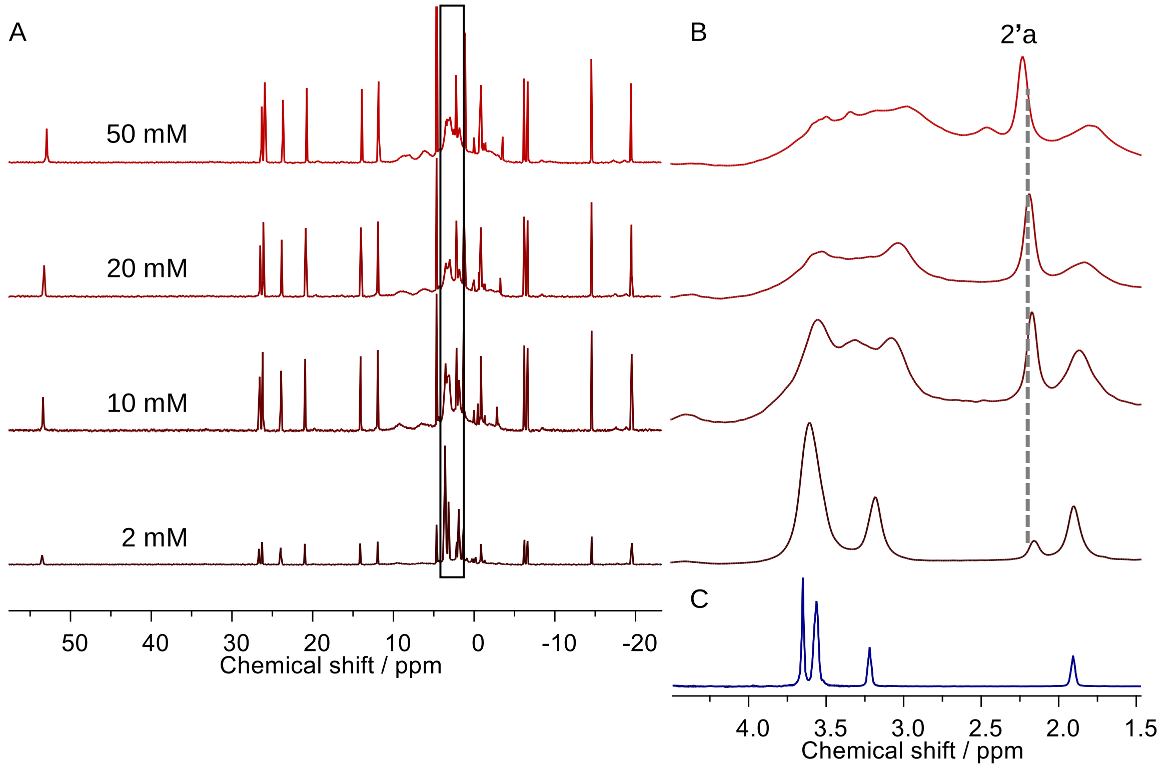

Figure S21: (A) ^{1}H NMR spectra of pD 5.1 D_2O solutions 2 mM in DEGTA and varying Eu(III) concentrations stated with the spectra. (B) Magnifications of a selected spectral region from (A). (C) Superposition of ^{1}H NMR blank spectra of the ligand pH-titration series obtained at pH 5.0 and 5.2.

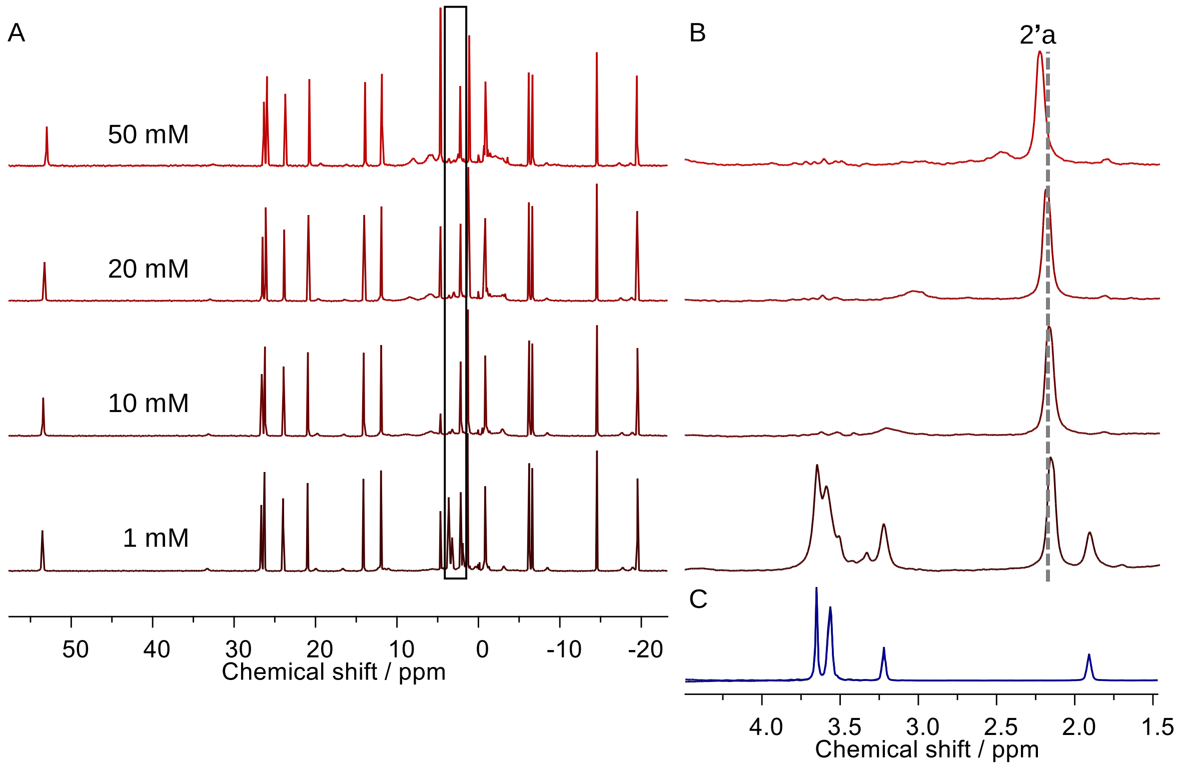

Figure S22: (A) ^{1}H NMR spectra of pD 6.1 D_2O solutions 2 mM in DEGTA and varying Eu(III) concentration as stated with the spectra. (B) Magnifications of a selected spectral region from (A). (C) Superposition of ^{1}H NMR blank spectra of the ligand pH-titration series obtained at pH 6.0 and 6.2.

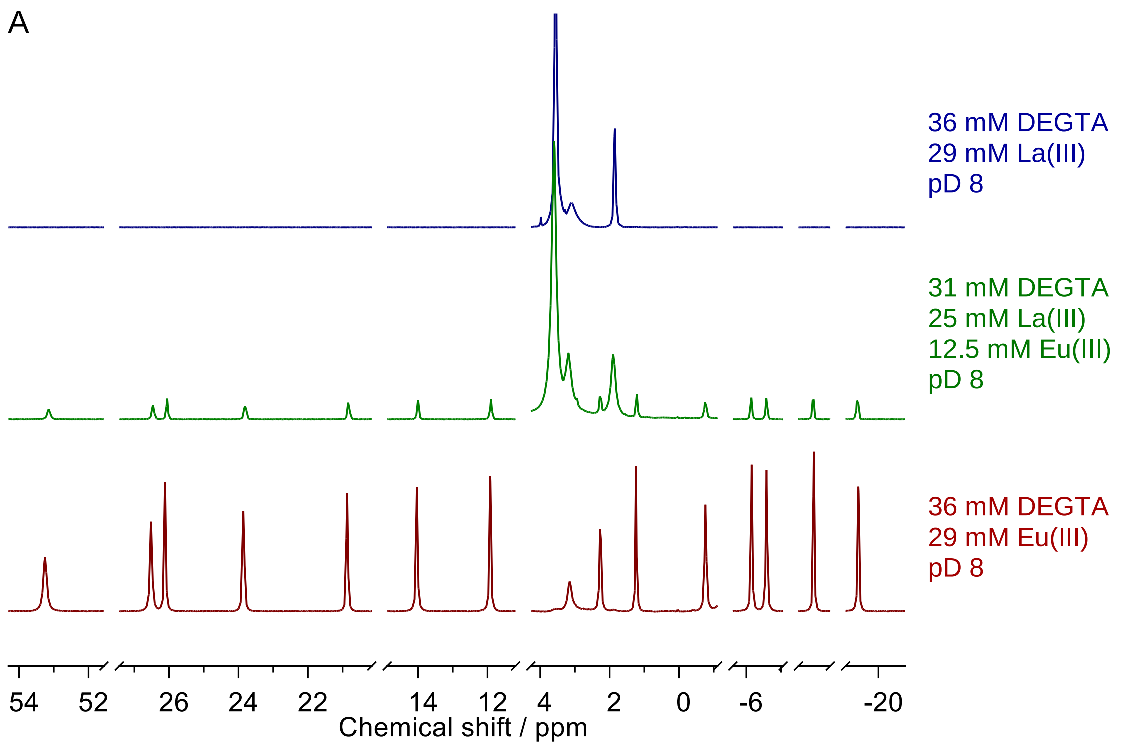

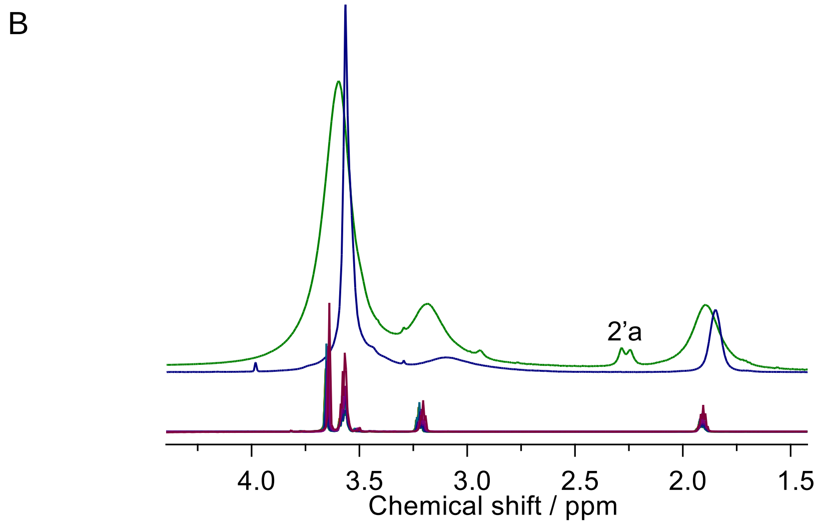

Figure S23: (A) ^{1}H NMR spectra of pD 8 D_2O solutions of composition as stated with the spectra. (B) Expansion of the top and middle spectrum from (A), i.e. those containing La(III) and La(III)/Eu(III) 2:1, respectively, along with superimposed ^{1}H NMR blank spectra in the range pH 6 through 8.5 at the bottom.

DFT calculations: Optimized structures and QTAIM analysis

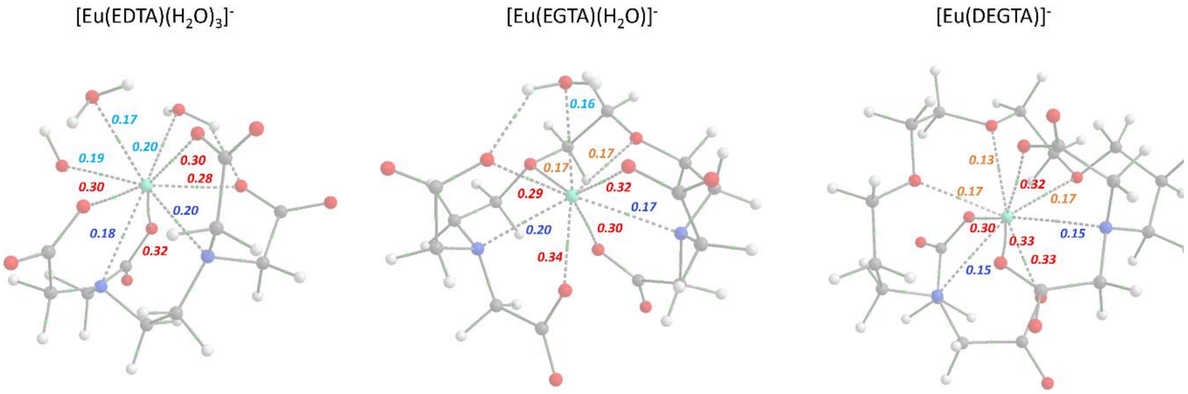

Figure S24: QTAIM-Analysis: Delocalization Indices of $[Eu(EDTA)(H_2O)_3]^-$ (left), $[Eu(EGTA)(H_2O)]^-$ (middle) and $[Eu(DEGTA)]^-$.

Table S3: Selected calculated distances (in Ångström) and delocalization indices (DI) between atoms from the DEGTA 1:1-complexes with La(III), Eu(III) and Cm(III). M stands for La, Eu or Cm, respectively.

	$[La(DEGTA)]^-$		$[Eu(DEGTA)]^-$		$[Cm(DEGTA)]^-$	
	bond length / Å	DI	bond length / Å	DI	bond length / Å	DI
M-O_{carb}	2.472	0.31	2.321	0.33	2.373	0.34
M-O_{carb}	2.476	0.30	2.346	0.33	2.384	0.32
M-O_{carb}	2.479	0.30	2.348	0.32	2.398	0.31
M-O_{carb}	2.502	0.28	2.349	0.30	2.420	0.30
M-O_{ether}	2.615	0.18	2.516	0.17	2.538	0.21
M-O_{ether}	2.661	0.17	2.565	0.17	2.599	0.20
M-O_{ether}	2.672	0.15	2.599	0.13	2.616	0.16
M-N	2.796	0.18	2.748	0.15	2.727	0.20
M-N	2.835	0.17	2.777	0.15	2.751	0.19

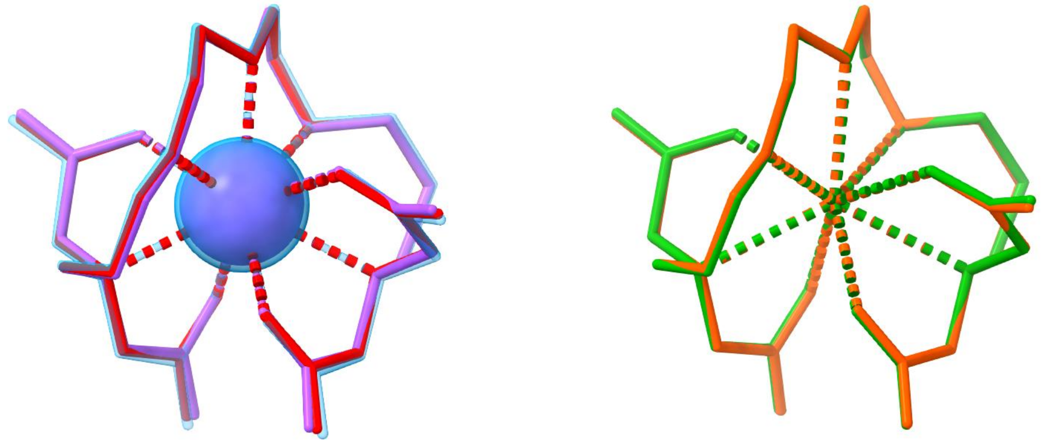

Figure S25: Structure overlay of $[La(DEGTA)]^-$ (transparent cyan), $[Eu(DEGTA)]^-$ (violet) and $[Cm(DEGTA)]^-$ (red) (*left*), and comparison of structures of $[Eu(DEGTA)]^-$ optimized with TURBOMOLE (green) and ORCA (orange) in the TZVPP basis (*right*).

DFT calculations: Harmonic IR-spectra

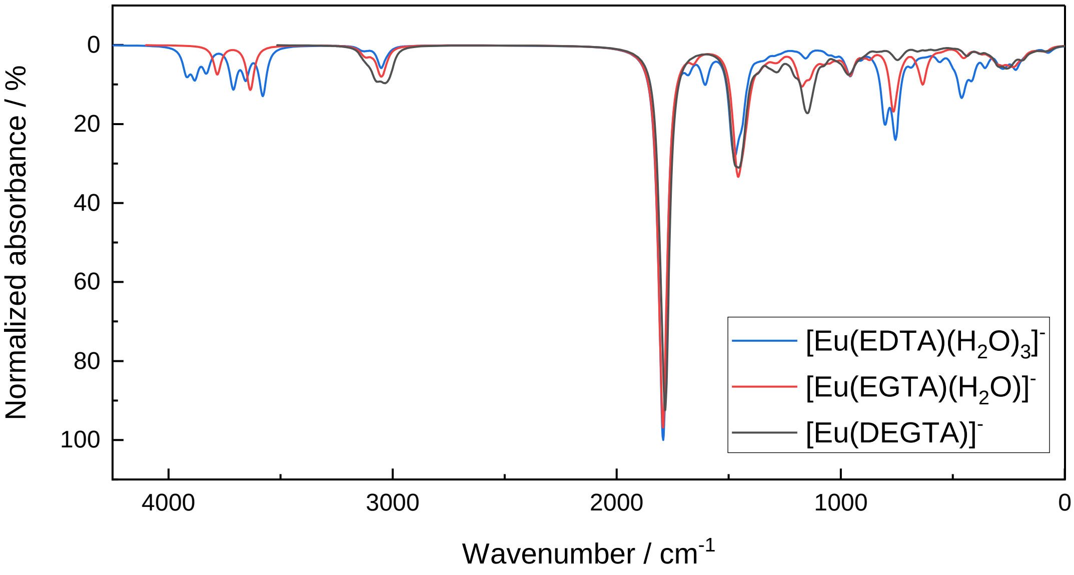

Figure S26: Simulated IR-Spectra of the complexes $[Eu(EDTA)]^{-}$, $[Eu(EGTA)]^{-}$, and $[Eu(DEGTA)]^{-}$ obtained from vibrational frequency analysis after optimization: Lorentzian line broadening, FWHM = 20 cm^{-1}, DFT parameters: def-SVP / PBE0 / ECP / COSMO(H2O). Characteristic frequencies: ca. 3500 cm^{-1} - 3900 cm^{-1}: O-H stretching, ca. 3020 cm^{-1} - 3080 cm^{-1}: C-H stretching, ca. 1790 cm^{-1}: COO stretching (ν_{as}), ca. 1600 cm^{-1}: H_2O bending (δ), ca. 1460 cm^{-1}: COO stretching (ν_s), ca. 1150 cm^{-1}: C-O stretching.

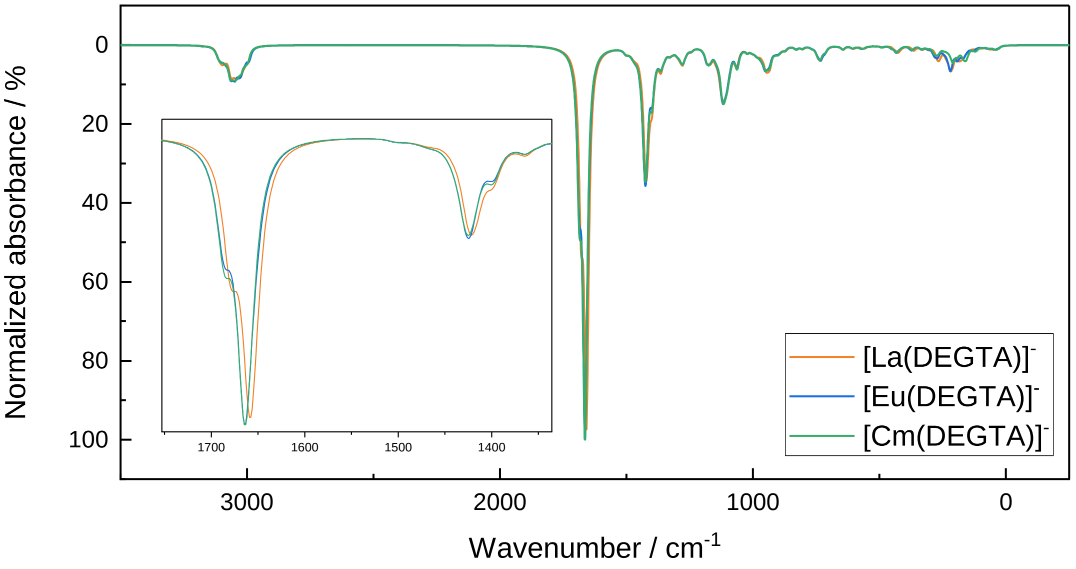

Figure S27: Simulated IR-Spectra of the complexes $[La(DEGTA)]^{-}$, $[Eu(DEGTA)]^{-}$, and $[Cm(DEGTA)]^{-}$ obtained from vibrational frequency analysis after optimization: Lorentzian line broadening, FWHM = 20 cm^{-1}, DFT parameters: X2C-TZVPALL+ SARC-DKH-TZVPP / PBE0 / no ECP / CPCM(H2O)

DFT calculations: ΔG comparison of low-lying conformers of [Eu(DEGTA)]⁻

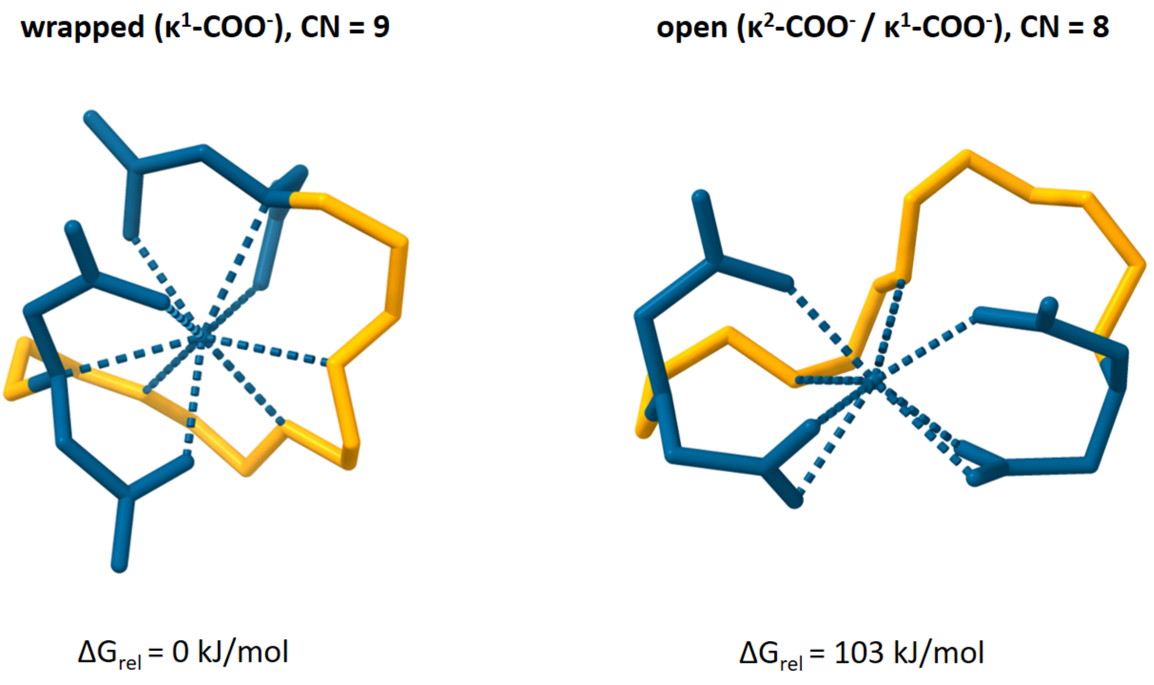

Figure S28: Wrapped, almost C_2 symmetrical form of the DEGTA-Eu complex (left) and its open 8-fold coordinated variant conformer (right), which is much higher in energy. The ether backbone is highlighted in yellow.

CASSCF calculations: Reconstruction of the PCS field of [Eu(DEGTA)]⁻

Using the example case of [Eu(DEGTA)]⁻, a State-averaged Complete Active Space Self-Consistent Field (SA-CASSCF) calculation was performed with the goal of extracting the magnetic susceptibility tensor as well as the electron spin density, to finally reconstruct the Pseudo-Contact Shift (PCS) field and the respective additional chemical shifts experienced by the protons, originating in the six unpaired electrons of the Eu center. As a preliminary step, the geometry was refined in the def-TZVPP basis in order to start from a similar basis set subsequently used for the more sophisticated CASSCF procedure. Here, all electrons are considered explicitly and scalar relativistic effects are introduced through the Douglas-Kroll-Hess[47][48] (DKH) formalism with appropriate basis sets (DKH-DEF2-TZVPP, Eu: SARC-DKH-TZVPP[49]). The chosen active space consists of the seven 4*f* orbitals of the Eu center. Considering all possible arrangements of the six unpaired electrons within the quasi-degenerate orbitals, following configuration state functions were included: 7 septets, 140 quintets, 588 triplets and 490 singlets. The CAS calculation was performed using ORCA 5.0.4[40]. The calculation of the PCS field uses the relation expressed in the Kuprov (Charnock, G. T. P.; Kuprov, I. A Partial Differential Equation for Pseudocontact Shift. *Phys. Chem. Chem. Phys.* **2014**, *16* (37), 20184−20189.) equation acknowledging the spatial distribution of the unpaired electron density and was performed using the MATLAB package Spinach (Hogben, H. J.; Krzystyniak, M.; Charnock, G. T. P.; Hore, P. J.; Kuprov, I. Spinach − A Software Library for Simulation of Spin Dynamics in Large Spin Systems. *J. Magn. Reson.* **2011**, *208* (2), 179− 194.). The obtained PCS field represented through iso-surface plots can be seen in Figure S29.

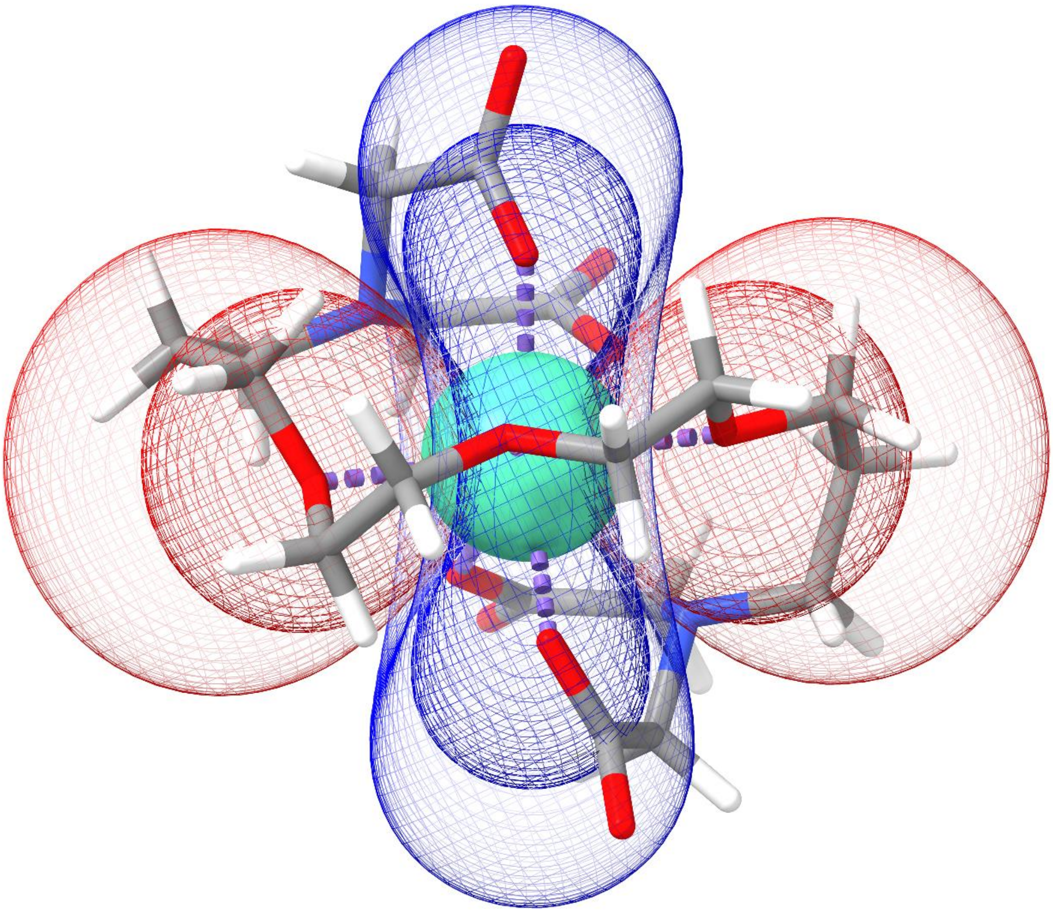

Figure S29: Optimized molecular structure of the [Eu(DEGTA)]$^{-}$ complex with iso-surface plots of the calculated PCS field (outer plot: ± 20 ppm, inner plot: ± 50 ppm, red: positive, blue: negative).
